# Sinomenine in cerebral ischemia: mechanisms, delivery strategies, and the emerging multifunctional biomaterials of the bone–brain axis

**DOI:** 10.3389/fmed.2025.1677685

**Published:** 2025-10-22

**Authors:** Xiang Li, Xinyi Zhang, Tingyu Wang, Hangyu Li, Yuxuan Li, Jianchun Liu, Yuqi Bai

**Affiliations:** ^1^Queen Mary School, Jiangxi Medical College, Nanchang University, Nanchang, China; ^2^School of Ophthalmology and Optometry, Nanchang University, Nanchang, China; ^3^School of Physics and Materials Science, Nanchang University, Nanchang, Jiangxi, China; ^4^School of Environment, Education and Development, The University of Manchester, Manchester, United Kingdom

**Keywords:** sinomenine, cerebral ischemia, drug delivery, neuroinflammation, bone–brainaxis

## Abstract

Cerebral ischemia, a leading cause of neurological disability and death, is characterized by reduced cerebral blood flow that induces hypoxia, neuronal injury, and irreversible brain damage. Its complex pathophysiology involves neuroinflammation, oxidative stress, glial cell activation, disruption of the neurovascular unit, and increasingly recognized bone–brain axis crosstalk. Sinomenine (SIN), a bioactive alkaloid derived from *Sinomenium acutum*, exhibits notable anti-inflammatory, antioxidant, and immunomodulatory properties, and has shown protective effects on the cardiovascular and nervous systems. However, the clinical application of SIN is limited by its poor pharmacokinetic properties, such as low oral bioavailability and a short half-life. To address these limitations, nanotechnology-based delivery systems have been designed to enhance its stability, brain-targeting ability, and therapeutic potential. Recent studies also highlight the potential of leveraging the bone–brain axis as a novel route for SIN delivery, offering enhanced targeting of ischemic brain tissue. This review synthesizes current evidence on the neuroprotective mechanisms of SIN, with particular focus on its modulation of the bone–brain axis and the advances in delivery technologies. Collectively, these insights support the therapeutic potential of SIN-based nano-delivery platforms targeting the bone–brain axis in the treatment of cerebral ischemia.

## 1 Introduction

Research on the use of traditional Chinese medicine, including single herbs, classical prescriptions, and isolated bioactive compounds for the prevention and treatment of central nervous system (CNS) disorders has been conducted for several decades. Accumulating evidence highlights their considerable therapeutic potential, with clinical benefits documented across a range of neurological conditions. For example, recent studies have shown that *Lonicerae Japonicae Flos* alleviates Alzheimer’s disease pathology through multiple targets and active constituents (1). Ginsenoside Rb1 has been reported to exert neuroprotective effects against cerebral ischemia–reperfusion injury, primarily through the regulation of antioxidant defenses and suppression of neuroinflammatory response (2). Similarly, Zhang et al. (3) demonstrated that mangiferin preserved neurological function and improved post-stroke cognitive impairment in I/R rats, primarily via regulation of disordered lipid metabolism. Sinomenine (SIN), a major bioactive alkaloid isolated from the root and stem of *Sinomenium acutum* Rehder and E. H. Wilson or its variant *Sinomenium acutum* var. *cinereum*, is the primary pharmacologically active component of this traditional Chinese medicinal herb. It has been historically used in the treatment of rheumatism and neuralgia and has a long history of clinical application in traditional medicines (4–6). The pharmacological activities of SIN, which include immunomodulatory and anti-inflammatory properties, are responsible for the significant therapeutic efficacy of SIN in the treatment of conditions such as rheumatoid arthritis, sciatic neuritis, and lumbalgia (4, 5, 7). Since its purification in the 1920s, SIN has also been shown to regulate histamine release, exert mild sedative and analgesic effects, and provide neuroprotective benefits. These pharmacological actions have been substantiated in various disorders, including ankylosing spondylitis and CNS diseases (4, 5, 8–13).

Stroke, particularly ischemic stroke which accounts for approximately 85% of all cases in China—is a major cause of death and long-term disability (14–16). In the event of a stroke, the depletion of adenosine triphosphate (ATP) triggers the ischemic cascade, which ultimately leads to the accumulation of calcium within the cells, the failure of the ion pump, and the glutamate-induced cell excitotoxicity (17). In addition to the response of the central neurons, the injured tissue also undergoes activation of the immune system. The neuronal cells in the brain are the cells that are most vulnerable to harm from ischemia (18). Damage associated molecular patterns (DAMPs) are molecules that are released by necrotic neurons and non-neuronal tissue. Examples of DAMPs include high-mobility group box 1 (HMGB1) and heat shock proteins (HSPs), which, together with other pro-inflammatory mediators, contribute to the disruption of the blood–brain barrier (BBB) (19). Once the BBB is breached, systemic inflammatory cells such as monocytes, neutrophils, and T cells continuously infiltrate the lesion, worsening the injury. In addition, multi-omics analyses revealed that dynamic alterations in cerebrospinal fluid lipid metabolism during the course of intracerebral hemorrhage from onset to reperfusion are closely associated with patient outcomes, suggesting that lipid metabolism may serve as a potential target for therapeutic intervention and prognostic evaluation (20).

Bone, as a fundamental structural tissue, has garnered increasing attention for its endocrine functions. The bone-brain axis, also known as the brain-bone axis, is a bidirectional regulatory interaction between the brain and bone that can be formed by a variety of methods, according to new research. The efferent neural system controls bone homeostasis and regeneration (21–23), However, the mechanisms behind the regulatory effects of bone on brain function are still being studied (24–26). According to recent research, bone can control peripheral organs’ growth and metabolism through bone-derived cell migration and the synthesis and secretion of several bioactive cytokines. Certain bone-derived cytokines and cells can traverse the BBB to reach the CNS (27).

**Figure 1:**
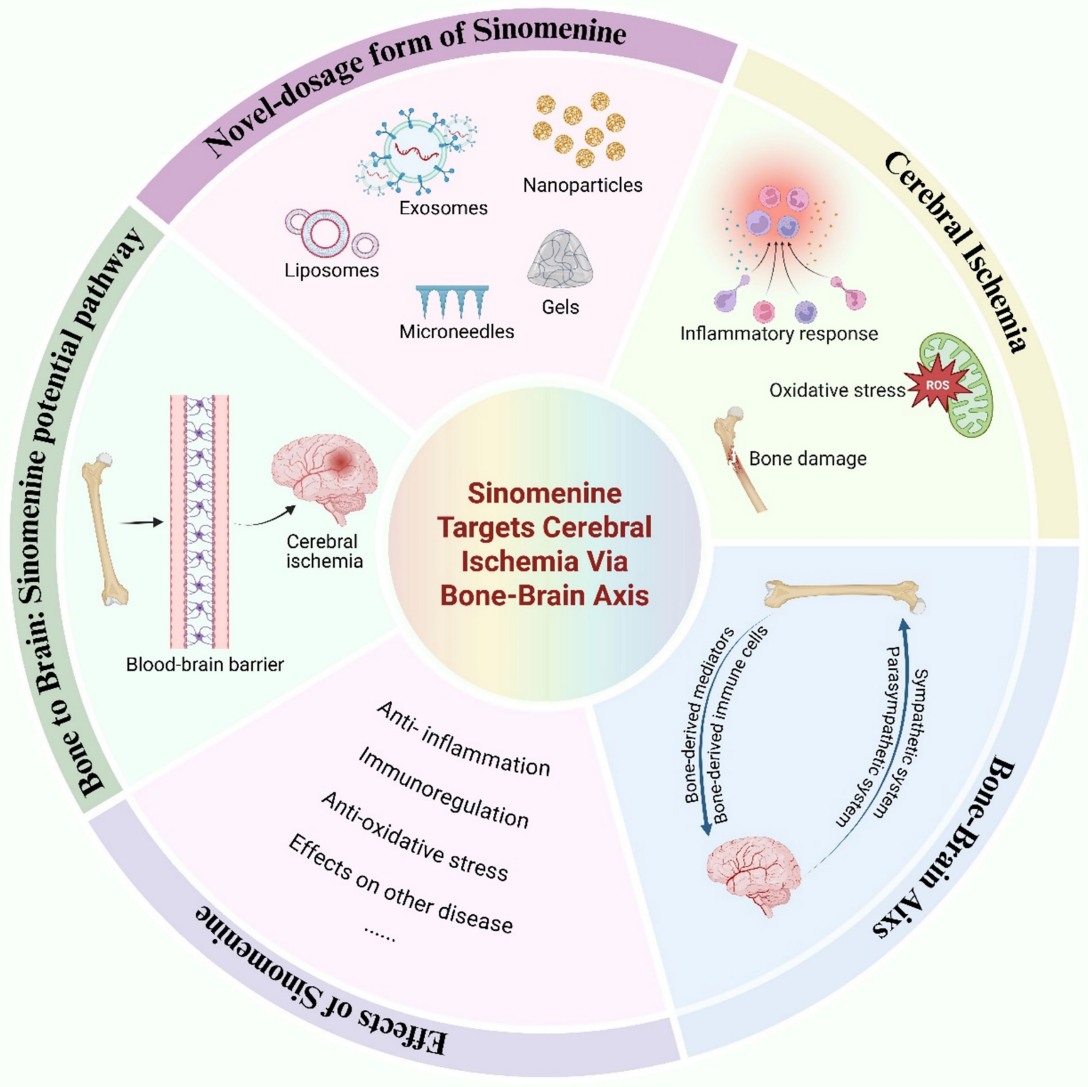
Targeting cerebral ischemia via the bone-brain axis: therapeutic potential of Sinomenine.

Given the multifaceted pharmacological actions of SIN and the emerging insights into the regulatory crosstalk between bone and brain, we propose a novel therapeutic paradigm that leverages the bone-brain axis for the targeted delivery of SIN in the treatment of cerebral ischemia. To illustrate the conceptual framework and hypothesized mechanisms underlying this approach, we present a graphical summary (Figure 1) outlining the pathophysiological processes of cerebral ischemia, the pharmacological effects of SIN, and its potential delivery via the bone-brain axis.

## 2 Pathophysiological mechanisms of neurovascular injury induced by cerebral ischemia

Studies have demonstrated that cerebral ischemia triggers a multifactorial and complex pathological process (28). Out of all of these mechanisms, inflammation is a significant contributor to the pathophysiology of ischemia. Nuclear factor-kappa B (NF-κB) and activator protein-1 (AP-1) are key transcriptional regulators of the inflammatory response, promoting the upregulation of pro-inflammatory cytokines such as interleukin-1β (IL-1β) and tumor necrosis factor-*α* (TNF-α). The following activation of matrix metalloproteinases (MMPs) and cyclooxygenase-2 (COX-2) is a consequence of this cascade (29). The activation of MMP-2, MMP-3, and MMP-9 compromises key structural components, including the basement membrane, tight junctions, and extracellular matrix, thereby in-creasing BBB permeability. This results in secondary effects, including leukocyte in-filtration, impaired transcellular transport, pericyte damage, expansion of the peri-vascular space, and astrocyte proliferation (30, 31). Additionally, chronic cerebral ischemia drives the sustained activation and proliferation of M1-type microglia and A1-type reactive astrocytes, which also increases the release of pro-inflammatory cytokines like IL-1β and TNF-*α*, as well as neurotoxic mediators (32–34). This microglial activation of microglia is potentiated by paracrine signaling (35). On the one hand, IL-1β exacerbates BBB disruption by downregulating tight junction protein Zonula Oc-cludens-1 (ZO-1), activating caspase-1 within the brain, and establishing a positive feedback loop that amplifies IL-1β production (36). On the other hand, the upregulation of TNF-*α* in endothelial cells promotes cytoskeletal rearrangement, thereby compromising tight junction integrity and disrupting the structural cohesion of the basement membrane (37). Moreover, TNF-α contributes to endothelial dysfunction by activating the NF-κB and mitogen-activated protein kinase (MAPK) signaling pathways, which in turn promote the transcription of additional pro-inflammatory cytokines and apoptosis-related genes. This cascade of events exacerbates endothelial cell activation and dysfunction (38–41).

Furthermore, activated astrocytes differentiate into neurotoxic A1 and neuroprotective A2 subtypes (42, 43). Under cerebral ischemic conditions, reactive astrocytes predominantly adopt the A1 phenotype, characterized by the release of pro-inflammatory factors and neurotoxic mediators. A1 astrocytes are distinguished by hypertrophy, reduced expression of essential ion channels and neurotransmitter receptors, and upregulated glial fibrillary acidic protein (GFAP) expression, ultimately disrupting the BBB (44). Recent studies have demonstrated that in the bilateral carotid artery stenosis (BCAS) mouse model of chronic cerebral ischemia, sham-operated controls were used for comparison, and a significant increase in GFAP^+^ astrocytes was observed within 28 days after surgery (45). This astrocytic activation was accompanied by pronounced demyelination and axonal injury in the white matter (45). Thus, microglia and astrocytes play key roles in the pathogenesis and progression of cerebral ischemia.

Cerebral ischemia also activates inflammasomes, thereby initiating an inflammatory response. Studies have shown that the NOD-like receptor thermal protein domain associated protein 3 (NLRP 3) inflammasome, in response to cerebral ischemia, is activated through the pathogen-associated molecular pattern (PAMP)/DAMP signaling pathway, which promotes the conversion of caspase-1, thereby accelerating the process of pyroptosis in neuronal cells (46). Additionally, persistent cerebral ischemia induces anaerobic metabolism in brain cells, leading to the onset of oxidative stress. Research indicates that following cerebral ischemia, antioxidant enzyme levels significantly de-crease, reactive oxygen species (ROS) production increases, and excessive deposition of amyloid-beta (Aβ) proteins occurs (47). Meanwhile, methyltransferase 3-mediated m6A modification promotes the degradation of synaptosomal-associated protein 29 mRNA, thereby impairing the restoration of autophagic flux, triggering mitochondrial crisis and excessive ROS accumulation, and ultimately exacerbating oxidative stress injury in the ischemic microenvironment (48). Consequently, these changes result in extensive oxidative damage to proteins, DNA, and other cellular components within the brain.

**Figure 2:**
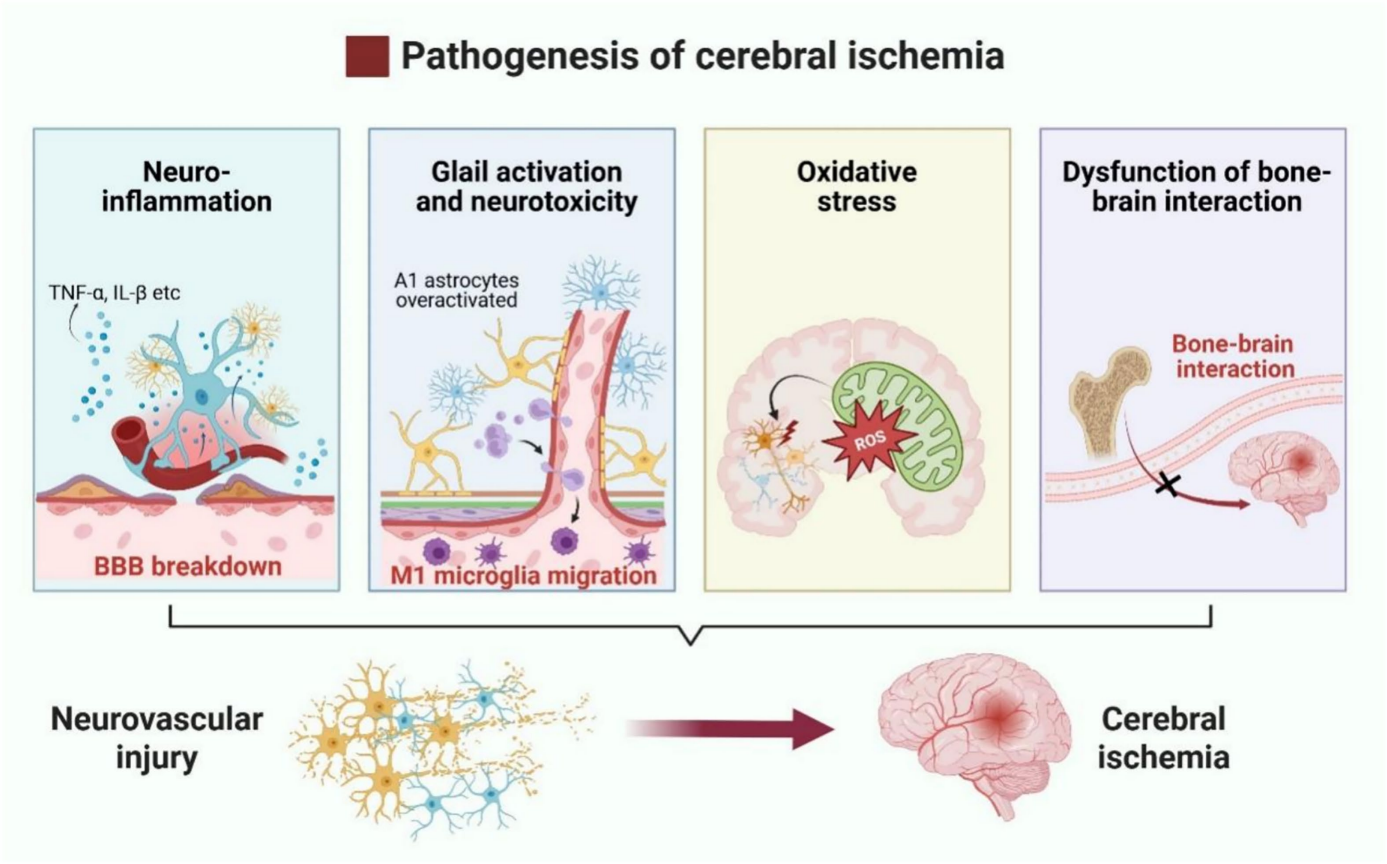
Pathophysiological mechanisms of neurovascular injury in cerebral ischemia. Neurovascular injury in cerebral ischemia is primarily driven by neuroinflammation, overactivation of pro-inflammatory glial cells, oxidative stress, and disruption of bone–brain axis interactions.

Notably, cerebral ischemia may interact with bone through multiple pathways. Stroke, a major risk factor for fragility fractures, increases the risk of post-stroke disability and mortality, with stroke survivors experiencing a seven-fold higher incidence of fragility fractures (49). Wang et al. (50) found that cerebral ischemia can activate the sympathetic nervous system, which in turn activates CD4^+^ T cells in the bone marrow. Upon activation, Receptor Activator of Nuclear Factor-κB Ligand (RANKL), is produced by CD4^+^ T cells, which attaches to the RANK receptor and triggers the NF-κB and MAPK signaling pathways. This activation promotes Nuclear Factor of Activated T-Cells Cytoplasmic 1 (NFATc1), −mediated osteoclast differentiation, ultimately enhancing osteoclastogenesis and bone resorption (51). Conversely, bone is also considered an endocrine organ (52), influencing the pathological state of cerebral ischemia through cytokine-mediated indirect mechanisms. Members of the transforming growth factor-β (TGF-β) class, bone morphogenetic proteins (BMPs) are signaling molecules released by osteoblasts (OBs) and osteoclasts (OCs) (53). BMPs primarily regulate bone formation and maintenance via Smad-dependent signaling and through crosstalk with other pathways such as MAPK, Wnt, Hedgehog, Notch, and FGF signaling (54). Ap-proximately 15 BMPs have been identified, with some being implicated in ischemic brain injury (55). For example, BMP7 is significantly upregulated following cerebral is-chemic injury, likely due to its neuroprotective effects. In a rat model of cerebral ischemia, Pei et al. (56) demonstrated that exogenous injection of BMP7 effectively mitigated neuronal death induced by ischemia, improved glutathione peroxidase (GSH-PX) and superoxide dismutase (SOD) activities, and mitigated neuronal death caused by ischemia, ultimately improving oxidative stress-induced brain damage. Similarly, BMP4/Smad1/5/8 signaling is enhanced in ischemic mice following isoflurane treatment (57), while BMP2 has been implicated in post-ischemic brain repair (58). Additionally, osteocalcin, a bone-derived hormone, has been shown to alleviate neuronal loss in acute ischemic stroke by inhibiting prolyl hydroxylase domain-containing protein 1(PHD1) and preventing the degradation of gasdermin D, thereby improving prognosis (59). These findings underscore the complex bidirectional interactions between bone and brain, revealing potential therapeutic targets for cerebral ischemia and other neurological disorders (Figure 2).

## 3 Bone-brain axis

The primary physiological function of bones has traditionally been viewed as protecting internal organs and facilitating movement. Given the structural characteristics of bones, they are often considered as the body’s framework, with little attention paid to their interactions with other organs. However, the emerging concept of the “bone-brain axis” has challenged this traditional view (21, 60), suggesting that the skeletal system and the CNS are interconnected entities with distinct functions within the body. There exists a complex interaction between the CNS and bone, with one key aspect being the neuroregulation of bone metabolism. Researchers have discovered that the CNS mediates neuronal circuits that directly regulate bone metabolism. Both autonomic and sensory nerve fibers can regulate the balance between OBs and OCs, thereby influencing bone remodeling (61). Sympathetic nerve fibers directly control OBs and OCs, affecting bone resorption and formation. This process is primarily mediated by the neurotransmitter norepinephrine (NE), which binds to β2-adrenergic receptors (β2-ARs) on OBs, triggering a signaling cascade (62) hat upregulates receptor activator of RANKL. RANKL subsequently promotes OC differentiation and activation, leading to increased bone resorption and structural degradation (63).

Leptin, a hypothalamic neuropeptide primarily secreted by adipocytes (64), plays a central role in regulating bone mass and has been shown to be closely associated with sympathetic nervous system (SNS) activity. Leptin directly affects bone tissue by promoting OB differentiation and mineralization of the bone matrix (61). Moreover, leptin can cross the BBB and bind to receptors in the arcuate nucleus of the hypothalamus, regulating neurotransmitters such as neuropeptide Y (NPY), cocaine-amphetamine regulated transcript (CART), serotonin (5-HT), and neuropeptide U (NPU), thereby in-directly influencing the bone-brain neural network (61). These changes lead to improved sympathetic outflow to the skeleton, followed by activation of OB β2-ARs, leading to increased bone resorption and decreased bone growth (65). Emerging evidence also suggests that the parasympathetic nervous system (PNS) can counterbalance the effects of the SNS on bone metabolism. Unlike the SNS, bone metabolic activity is primarily controlled by acetylcholine (ACh) through nicotinic acetylcholine receptors (nAChRs) on OBs. This interaction activates OBs, promoting their proliferation and differentiation, and thus influencing bone anabolism (65). Other neurotransmitters and neuropeptides, including glutamate, vasoactive intestinal peptide (VIP), *γ*-aminobutyric acid (GABA), calcitonin gene-related peptide (CGRP), growth hormone releasing peptide (GHRP), corticotropin-releasing factor (CRF), and CART (61, 62, 65–67), have also been implicated in diverse signaling pathways that ultimately govern bone metabolism and synthesis.

Interestingly, bone also can produce regulatory factors that influence neural activity (68). Bone-derived mediators are released from bone cells and bone marrow, crossing the BBB to regulate various aspects of CNS function, including memory, mood, and cognitive abilities (65). This interaction suggests that bone can serve as a crucial regulator of brain development, function, and pathophysiology (69). One of the most crucial bone-derived components for controlling brain activity is osteocalcin, a non-collagenous bone matrix protein that is exclusively made by OBs and is essential for preserving healthy bone mineralization. By promoting the synthesis of monoamine neurotransmitters, inhibiting the production of GABA, and preventing apoptosis in hippocampus neurons, osteocalcin can pass the BBB (52). Increasing numbers of studies have explored the regulatory effects of osteocalcin on the brain. For instance, Bradburn et al. (70) con-ducted a study involving 225 elderly individuals and 134 young adults that plasma osteocalcin levels were positively correlated with cognitive function and executive ability in the elderly. Oury et al. (71) demonstrated that osteocalcin plays a crucial role in promoting postnatal neurogenesis and enhancing memory, while also exerting anxiolytic and antidepressant effects. Furthermore, maternal osteocalcin can cross the placenta, supporting fetal brain development, including the maturation of spatial learning and memory functions. Chang et al. (72) found that osteocalcin alleviated pathological conditions in Alzheimer’s disease (AD) model mice by reducing Aβ burden and enhancing the glycolysis of astrocytes and microglia, providing new insights for AD treatment. Moreover, osteocalcin inhibits cholinergic activity in postganglionic parasympathetic neurons through its receptor, G protein-coupled receptor (GPCR) 6a, thereby enhancing the acute stress response (ASR) in animals, suggesting its role as a stress hormone regulating neural activity under danger (73).

AAs research advances, additional bone-derived proteins with neuroregulatory functions are being identified. Osteopontin, for example, is involved in both inflammatory responses and bone remodeling and metabolism, and it also affects the nervous system, playing a dual role in tissue damage and repair. By inhibiting the Wnt/β-catenin signaling pathway, sclerostin prevents the production of new bone, promoting bone resorption, and potentially impairing synaptic plasticity and memory. Both osteopontin and sclerostin exhibit potential as novel biomarkers for neurodegenerative diseases (74). Osteocytes and osteoblasts (OBs) secrete fibroblast growth factor 23 (FGF23), a bone-derived hormone that may similarly influence behavior and brain function. Studies have shown that FGF23 reduces the expression of brain-derived neurotrophic factor (BDNF) and other synaptic proteins, thereby impairing synaptic plasticity and cognitive performance. Elevated FGF23 levels have been associated with increased anxiety- and depressive-like behaviors, as well as impairments in memory and spatial learning (65). Collectively, these bone-derived molecules significantly impact the nervous system, not only by influencing bone remodeling but also by modulating neurotransmitter release and neuronal activity, ultimately shaping mood and cognition. This connection offers new perspectives for understanding the relationship between bone function and brain health.

Beyond neuroendocrine interactions, the bone-brain axis also encompasses com-plex immune crosstalk. Bone marrow-derived immune cells have been shown to play neuroprotective, neurorepair, and neuroplastic roles in brain diseases (75). These cells can migrate into brain tissue through various mechanisms (76). A recent study labeled bone marrow cells residing in the skull and tibia of mice with spectral membrane dyes as cellular trackers. Remarkably, they discovered vascular channels connecting the brain and bone marrow. Myeloid cells migrate through these microchannels to inflamed brain tissue, which are located across the skull’s endothelial layer, thereby linking the skull’s bone marrow cavity with the dura mater (77). Macrophages, a critical myeloid immune cell type, also play an essential role in the brain immunity. Reparative macrophages secrete factors such as osteocalcin, insulin-like growth factor 1 (IGF-1), platelet-derived growth factor, BMPs, and growth differentiation factor 15 (GDF-15), all of which support neurogenesis and tissue reconstruction. Additionally, macrophages produce angiogenic factors that promote collateral artery growth, and depletion of these cells has been as-sociated with increased bleeding incidents (78). T lymphocytes are central to the immune response in the brain. Regulatory T cells (Tregs) promote neural tissue repair by suppressing excessive astrocyte activation and promoting myelin regeneration (79). Furthermore, Treg-derived OBs enhance microglial repair activity via integrin receptors on microglial cells, thus improving white matter integrity and preserving neural function in the long term (80). Modulating peripheral immune cell interactions with the CNS presents a promising therapeutic avenue for enhancing recovery from brain injury.

**Figure 3:**
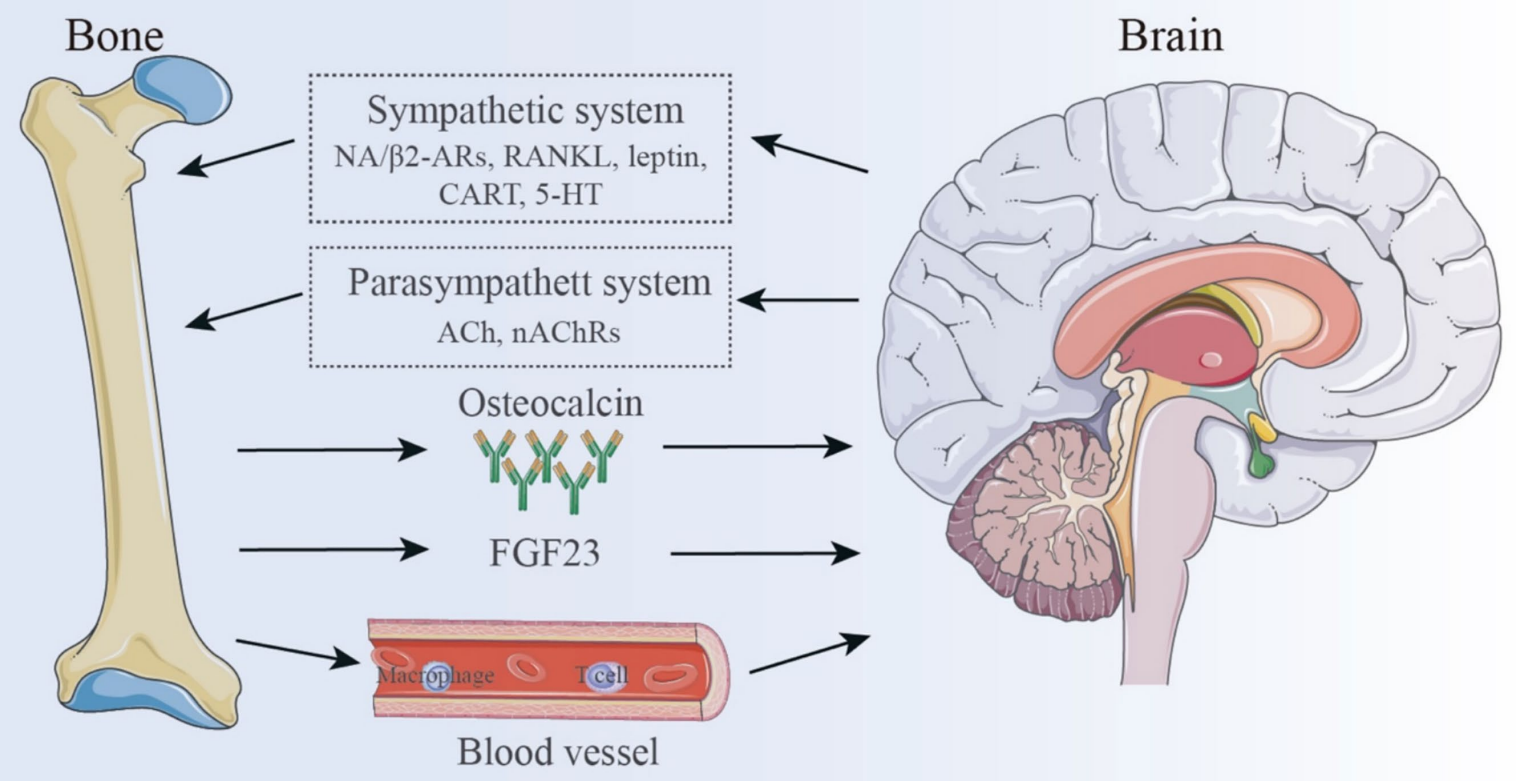
The pathological relationship between cerebral ischemia and the bone-brain axis.

In conclusion, the bone-brain axis is a highly integrated system governed by intricate regulatory mechanisms. This emerging field of research has illuminated the multifaceted roles of skeletal tissues in neuroregulation, metabolic control, and immune-inflammatory processes. As illustrated in Figure 3, the pathological interplay between cerebral ischemia and the bone-brain axis suggests that this axis may serve as a novel therapeutic target for neurodegenerative and cerebrovascular diseases. These in-sights not only offer fresh perspectives for early neurological intervention, but also pave the way for personalized treatment strategies and improved patient outcomes.

## 4 Pharmacokinetics of SIN

SIN, first isolated by Ishiwari from *Radix Sinomenii* in the 1920s, is a morphinan alkaloid with the chemical name (9α,13α,14α)-4-hydroxy-3,7-dimethoxy-17-methylmorphinan-6-one. Structurally, it consists of a hydrogenated phenanthrene core linked to an ethylamine bridge. SIN manifests as a crystalline powder, either whitish or pale-yellow, characterized by the molecular structure C₁₉H₂₃NO₄ and a comparative molecular mass of 329.38. This substance melts at 161 °C and dissolves in ethanol, acetone, chloroform, and diluted alkali, yet shows limited solubility in water, ether, and benzene.

SIN, mainly employed in managing rheumatoid arthritis and joint discomfort, has a broad range of pharmacological effects, encompassing anti-inflammatory, antioxidant, immunomodulatory, sedative, and pain-relieving impacts. Studies have shown that SIN reduces oxidative stress and protects against organ damage by downregulating inflammatory markers such as MDA and hydrogen peroxide (H₂O₂) in rat lungs subjected to chronic intermittent hypoxia-induced lung injury (81). Additionally, studies have shown that SIN initiates autophagy in human glioblastoma cells by stimulating ROS production, stopping the cell cycle during the G0/G1 phase, and reducing cell movement and invasion. It is recognized that the hydrochloride salt form suppresses NF-κB activation, hinders MMPs (MMP-2 and MMP-9) synthesis, and reverses the transition from epithelial to mesenchymal traits in cancerous cells. The results imply that SIN could be effective in preventing the spread of tumor cells during glioblastoma therapy (82). By inhibiting Matrix Metalloproteinase-2/−9 and preventing the transition from epithelial to mesenchymal cells, SIN Hydrochloride prevents the growth of human glioblastoma cells. In further studies, lipopolysaccharide (LPS)-induced changes in keratinocyte viability, apoptosis, and increased expression of inflammatory cytokines such as TNF-*α*, IL-6, and IL-8 can be reversed by SIN intervention. The neuroprotective effects of SIN have also been extensively demonstrated in preclinical studies of neuro-logical diseases. According to both *in vitro* and *in vivo* research, SIN can inhibit or block a variety of signaling pathways that contribute to the development of neurological disorders. It also shows neuroprotective effects by reducing inflammation, reducing oxidative stress, and inhibiting apoptosis (83, 84). As a multipurpose neuroprotective agent, SIN has promise for the treatment and prevention of neurological disorders.

The pharmacokinetics and tissue distribution of SIN have been extensively investigated in rats. Following a single oral administration of 25 mg/kg SIN hydrochloride, female Sprague–Dawley rats exhibited significantly greater systemic exposure than males, with higher AUC₀–∞ values (13,430.6 ± 2,781.2 h·ng/mL vs. 7,221.3 ± 2,133.5 h·ng/mL, *p* < 0.01) and elevated Cmax (1,623.5 ± 299.3 ng/mL vs. 1,138.2 ± 213.7 ng/mL, *p* < 0.05). In addition, females showed a longer elimination half-life (6.2 ± 1.4 h vs. 4.5 ± 1.0 h) and slower clearance (1.9 ± 0.5 L/h/kg vs. 3.4 ± 0.8 L/h/kg, *p* < 0.01) compared with males (85). Consistently, SIN concentrations in the visceral organs of female rats were markedly higher than those of males, confirming pronounced sex-related differences in its pharmacokinetic and tissue distribution profiles (85). These findings suggest that sex should be considered when evaluating the therapeutic application of SIN. As an alkaloid, SIN readily forms water-soluble salts upon reaction with acids, while its lipophilic nature facilitates gastrointestinal absorption through passive diffusion. Research on tissue distribution indicates that 45 min post-administration (oral dose 90 mg/kg), the levels of SIN in various rat tissues, ranked from highest to lowest, were detected as: kidney, liver, lung, spleen, heart, brain, and testicles (86). After 90 min, there was a marked reduction in SIN concentrations across all organs, with the liver and kidneys maintaining relatively higher levels, suggesting these as key organs for metabolism and elimination. The protein binding assay showed SIN (at ~4 μg/mL in rat or rabbit plasma) has a binding rate to albumin exceeding 60%, while binding to *α*-1-acid glycoprotein is only ~33% (86).

**Figure 4:**
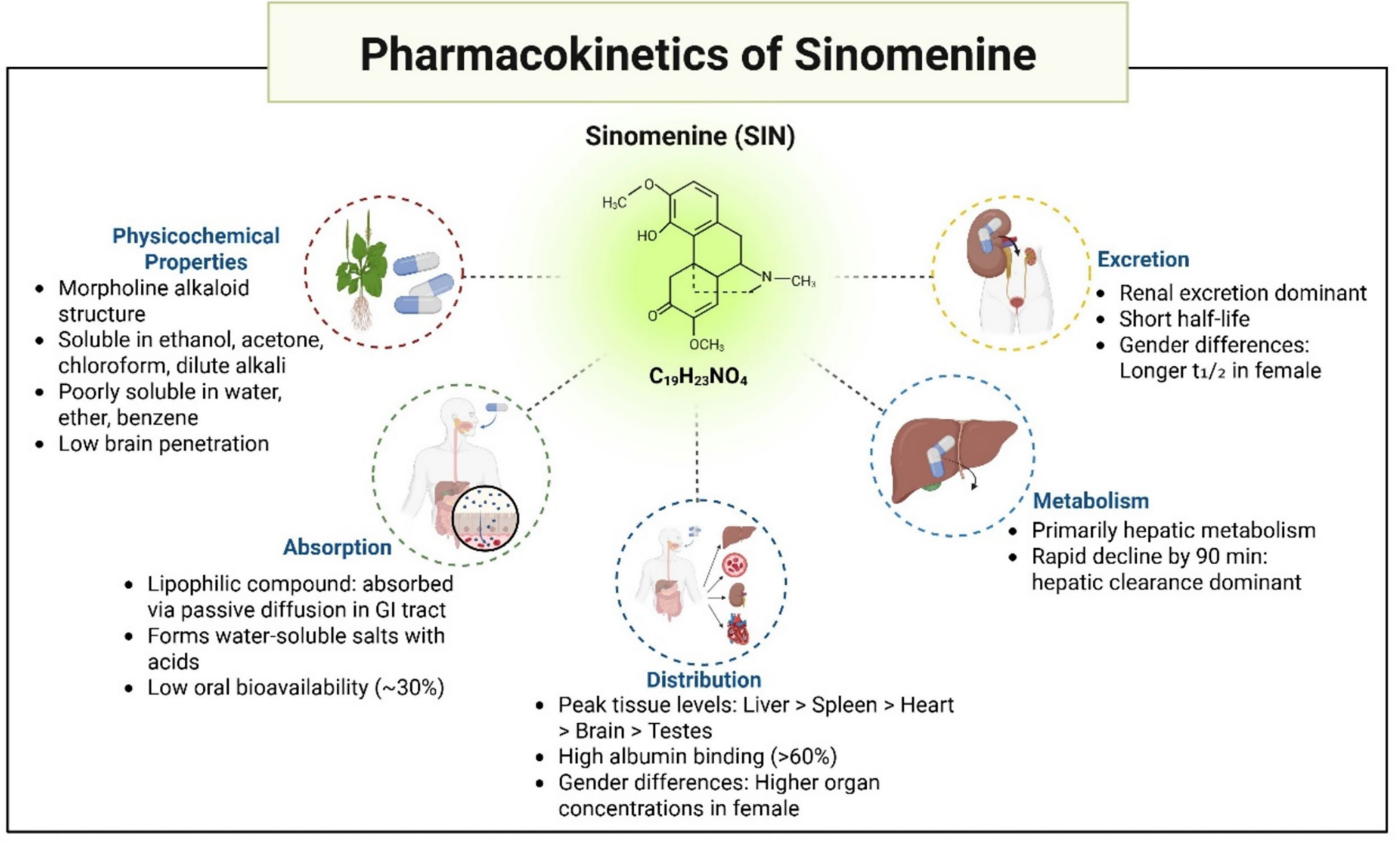
Pharmacokinetics and physicochemical properties of sinomenine.

Although SIN shows potential in pharmacology, its practical use in clinical settings is constrained due to its brief elimination half-life and minimal oral bioavailability, hovering around 30%. These factors somewhat limit its clinical application. Despite the various pharmacological effects of SIN in treating cerebral ischemia, its short half-life and low bioavailability increase the risk of adverse reactions. Therefore, the development of new drug delivery systems for SIN, aimed at achieving sustained release, reducing clinical dosages, increasing drug concentrations in the brain, and improving therapeutic outcomes, is of significant importance. These developments might offer a new approach in pharmacology for addressing cerebral ischemic conditions (Figure 4).

## 5 Pharmacological activities of SIN

**Figure 5:**
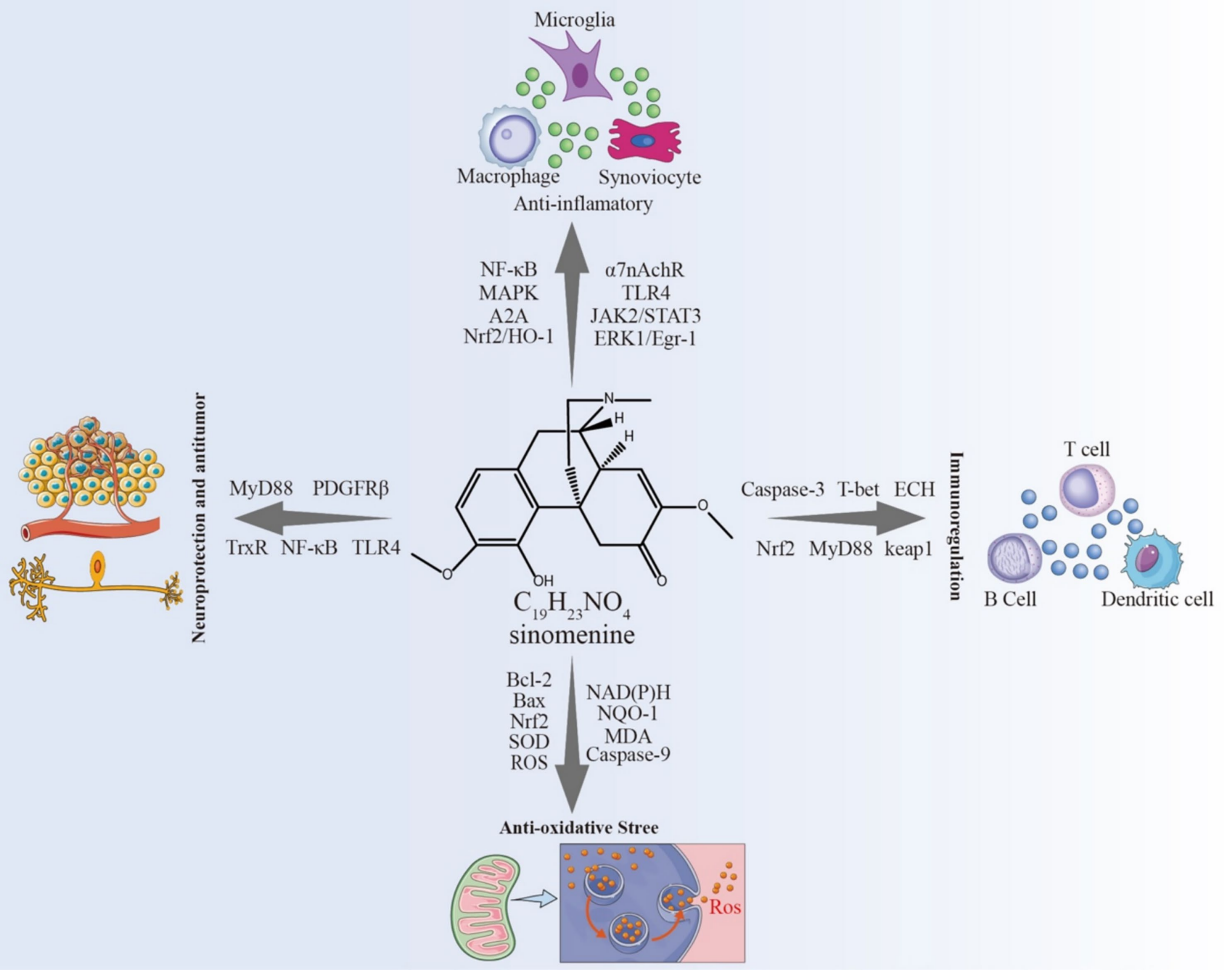
The pathological relationship between cerebral ischemia and the bone-brain axis.

Recent studies improved understanding of the cardiovascular protective effects of SIN, highlighting its roles in anti-inflammation, anti-oxidation, and endothelial protection. To reflect these advances, Table 1 presents an updated and comprehensive summary of the pharmacological actions of SIN in various cardiovascular disease models, incorporating findings from *in vitro* and *in vivo* studies (Figure 5).

**Table 1:** Summary of the cardiovascular protective effects exerted by SIN.

Experimental system	Model type	Mechanism insights	Dose/Concentration	Key findings	Validation methods	Year	Ref.
Anti-arrhythmia	I/R injury rats model	↓: PAF, ET-1, TBX2, TF, Fbg, PAI-1, Ck-MB, Ck, Tnl, MDA, LDH, AST, hs-CRP, MCP-1, TNF-*α*, IL-1β, IL-6 ↑: SOD、GPx、CAT	5, 10, 20 mg/kg	↓: Duration, incidence of ventricular fibrillation, ventricular ectopic beat, ventricular tachycardia, arrhythmia score, myocardial infarct area, platelet aggregation parameters	ECG analysis, biochemical assays, histopathology, ELISA	2022	(191)
Ameliorates cardiac hypertrophy	Ang II-treated H9C2 cells, isoproterenol-treated rat	↓: Cell surface area, ANP, BNP, β-MHC, cell apoptosis rate, caspase3, Bax, MDA, ROS ↑: Bcl-2, Nrf2, HO-1	40, 80 mg/kg	↓: HW index, LVW index	Western blot, RT-qPCR, TUNEL staining	2021	(192)
Anti-atherosclerosis	Atherosclerosis model	↓: Total cholesterol, triglyceride, LDL, VLDL, ET-1, hs-CRP, TXB2, cTnI、LDH, CK-MB, total protein, HMG-CoA/Mevalonate ratio, collagen, calcium, FFA, MDA, IL-1α, IL-1β, TNF-*α*, IL-6, IL-17, MCP-2, MCP-3, VCAM-1, ICAM-1 ↑: HDL, NO, CAT, SOD, GPx, GR, IL-10	5, 10, 20 mg/kg	↓: Body weight, water and food intake	Lipid profile analysis, histopathology, ELISA	2024	(193)
Vascular relaxation	Spontaneously hypertensive rats, A7r5 rat aortic smooth muscle cell line	↓: Ca ^2+^ ↑: Bcl-2, Nrf2, HO-1	2.5, 5, 10 mg/kg	↓: Systolic blood pressure ↑: vascular relaxation	↑: Vascular relaxation Blood pressure monitoring, vascular tension measurement, Western blot	2007	(194)
Protection against myocardial ischemia reperfusion injury	Myocardial ischemia–reperfusion injury mice	↓: CK, LDH, caspase-3, caspase-9, Bax, MDA, ROS, DHE, IL-1β, TNF-α, IL-6 ↑: EF, Bcl-2, GSH, T-AOC	80 mg/kg	↓: The ratio of infarct area to area at risk, the ratio of infarct area to left ventricle, TUNEL-positive cells	↑: Cardiac function TTC staining, echocardiography, TUNEL assay, oxidative stress markers	2022	(118)
Improve heart failure	Stress load-induced model of heart failure in mice	↓: ANP, type I and III collagen ↑: IL-10/IL-17	120 mg/kg	↓: LVEDd, LVEDs, LVPWd, inflammatory cell infiltration ↑: ejection fraction, shortened score	Echocardiography, Masson’s trichrome, ELISA	2020	(195)
Protect against sepsis-induced myocardial injury	Sepsis model	↓: IL-1β, TNF-α, IL-6, IFN-*γ*, CXCL8, JNK1, JAK1, JAK2, STAT3, NF-κB, cleaved-caspase3/caspase3, LVID, LDH, CK-MB, cMLC1, cTnI ↑: EF	50, 100 mg/kg	↓: Apoptosis of cardiomyocytes	Flow cytometry, Western blot, echocardiography	2023	(196)
Treatment of vascular proliferative disease	Rat VSMCs, carotid artery wire injury model	↓: MAPK signalling, AKT/GSK3β signalling, STAT3 phosphorylation, PDGFR-β phosphorylation ↑: SM α-actin, smoothelin, SMA	150 mg/kg	↓: VSMC proliferation, VSMC cell cycle progression, VSMC migration, artery injury-induced proliferative response, VSMC phenotype dedifferentiation	Immunohistochemistry, flow cytometry, Western blot	2013	(197)
Reduce myocardial infarction	Myocardial infarction model	↓: LDH ↑: EF, FS	50 mg/kg	↓: Myocardial infarction area	TTC staining, echocardiography	2022	(198)
Treatment of rheumatic carditis or rheumatic heart disease	HUVEC	↓: VCAM-1		NR	Flow cytometry, RT-qPCR	2007	(199)
Alleviated vascular calcification	Uremic rats	↑: miR-143-5p, mmu-mir-143-5p	40 mg/kg	↓: Vascular calcification	Alizarin Red staining, RT-qPCR, Western blot	2025	(200)
Ameliorate vascular calcification	Uremic rats, primary rat VSMCs	↓: NLRP3, Caspase-1, GSDMD, AEG-1	30 mg/kg	↓: Calcified area, vascular calcification	Von Kossa staining, Western blot	2025	(201)
Treatment of vascular proliferative disease	VSMC	↓: Phosphorylation of ERK1/2 and p38, Akt, GSK3β, STAT3, and PDGFR-β.	150 mg/kg	↓: Formation of neointima and number of PCNAP cells.	Immunofluorescence, Western blot	2013	(197)
Improving renal function	HRGECs	↑: Occludin, Nr. ↓: RhoA/ROCK signal transduction activation, abnormal occlusive protein distribution reversion, RhoA/ROCK, Cell permeability, and ROS.			Transwell permeability assay, Western blot	2016	(202)
Neuroprotection	Middle cerebral artery occlusion mice model	↓: NLRP3, cleaved caspase-1, cleaved caspase-3, IL-1β, IL-6, IL-18, TNF-α ↑: EF, FS	10 or 20 mg/kg	↓: Infarction volume, brain water content, neuronal loss and apoptosis, neurological deficiency, activation of astrocytes and microglia	TTC staining, TUNEL assay, behavioral tests	2016	(83)
Anti-inflammatory and cerebral protective functions	Middle cerebral artery occlusion model mice, BV2 cells	↓: TNF-α, IL-1β, SOD, GPx, IL-6, NOS2, Arg-1, IL-10, inhibition of OGD-induced IκBα phosphorylation and NF-κB p65 nuclear translocation ↑: Nrf2, HO-1, NQO1, NOS2, Arg-1, IL-10	20 mg/kg	↓: Brain water content ↑: Nrf2	Immunofluorescence, Western blot	2021	(203)
Suppresses neuroinflammation	Middle cerebral artery occlusion mice model, primary astrocyte	↓: Astrocytic activation, STAT3 phosphorylation, IL-1β, IL-6, IL-18, TNF-α, nuclear translocation of CRYAB ↑: DRD2, αB-crystallin, activation of STAT3	10, 20 mg/kg	↓: Infarction volume, brain water content ↑: alleviated neurological impairment	Western blot, ELISA	2016	(97)
Attenuates brain injury in intracerebral hemorrhage	ICH mouse model, BV2 cells	↓: IL-1β, IL-6, TNF-α, ROS, NF- κB, microglial migration	20 mg/kg	↓: Brain water content, neurological deficit scores	Behavioral tests, ELISA, immunohistochemistry	2014	(204)
Anti-inflammatory	Subarachnoid hemorrhage model	↓: Cleaved caspase-3, Bax, NF-κB, IL-1β, IL-6 ↑: Nrf2, HO-1, NQO1, Bcl-2	50 mg/kg	↓: Brain water content, early brain injury, neuronal apoptosis, neuronal degeneration, microglial activation, nrf2 expression, nuclear translocati ↑: neurological functions	TUNEL assay, Western blot, MRI	2023	(84)
Attenuates inflammatory injury	Autologous blood models, hippocampal tissue	↓: Matrix metalloproteinase 3/9, iNOS, IL-1β, TNF-α ↑: IL-10, Arg-1	20 mg/kg	↓: Brain water content, neurological impairment, microglia mediated toxicity to neurons, inflammation ↑: microglia m2 polarization	Immunofluorescence, ELISA	2016	(205)
Protects against ischaemic brain injury	Middle cerebral artery occlusion mice model, PC12 cells	↓: LDH, Bax, caspase-3, phosphorylation of CaMKII, L-type calcium currents, ASIC1a currents, KCl and acidification-mediated increase in [Ca^2+^]_i_ ↑: Bcl-2/Bax	10, 30 mg/kg	↓: Ischaemic sections	Electrophysiology, TTC staining, Western blot	2011	(144)
Anti-oxidative stress	PC12 neuronal cells	↓: MDA, Nrf2, activation of NOX ↑: GSH, SOD		↓: H2O2-induced cytotoxicity, oxidative injury, H2O2-induced cytotoxicity	MTT assay, ROS detection, Western blot	2017	(206)

### 5.1 Anti-inflammatory effects

NF-κB is a key nuclear transcription factor in eukaryotic cells. In most quiescent cells, NF-κB exists in an inactive complex in the cytplasm through its interaction with IκB. Upon stimulation by hypoxia, oxidative stress, or pro-inflammatory cytokines, IκB undergoes degradation, releasing NF-κB to translocate into the nucleus and induce transcriptional activity (87). Extensive research highlights the pivotal role of NF-κB in inflammatory diseases, with therapeutic interventions aimed at inhibiting NF-κB signaling showing potential for disease modulation. SIN achieves its anti-inflammatory properties by inhibiting the binding of NF-κB, consequently reducing the expression of inflammatory elements at the mRNA stage (88). Study by Xu et al. (89) in animal models confirmed that SIN attenuates inflammation by inhibiting both NF-κB and MAPK signaling pathways, leading to the downregulation of pro-inflammatory cytokines, such as IL-6 and TNF-*α*. Additionally, Yi et al. (90) reported the effects ofSINSIN on fibroblast-like synoviocytes (FLS) in adjuvant-induced arthritis (AIA) rat models. The research indicated that SIN amplifies the expression of adenosine A2A receptors in AIA rats and FLS via the α7 nicotinic acetylcholine receptor (α7nAChR) pathway, concurrently suppressing NF-κB activation, thereby mitigating arthritis. α7nAChR, an integral part of the cholinergic anti-inflammatory pathway (CAP), controls inflammation through acetylcholine signaling mediated by the vagus nerve. Remarkably, the elimination of α7nAChR nullified SIN’s inhibition of NF-κB activation, indicating a reliance on *α*7nAChR for its anti-inflammatory effects. Additionally, SIN influences the nuclear factor erythroid 2-related factor 2 (Nrf2)/ heme oxygenase-1 (HO-1) pathway, leading to the suppression of NF-κB activity in mouse chondrocytes, thereby curbing inflammatory reactions and the breakdown of extracellular matrix elements.

SIN influences this route through the suppression of CD14/ Toll-like receptor 4 (TLR4) and Janus Kinase 2 (JAK2)/ STAT3 signaling pathways, leading to a decrease in TNF-*α*, MCP-1, MIF, and MMP-9 levels in RAW 264.7 cells stimulated by LPS, and concurrently hindering the secretion of calcium ions within cells (91). Within AIA rats, TNF-α enhances FLS growth, increases α7nAChR levels, and stimulates the ERK/Egr-1 pathway, while SIN mitigates these impacts by reducing FLS growth, α7nAChR levels, and ERK/Egr-1 pathway activation (92). Research on lung cancer revealed that SIN suppresses α7nAChR, pERK1/2, ERK1/2, and the transcription factors TTF-1 and SP-1, simultaneously boosting Egr-1 expression for its cancer-fighting properties (93). Ad-ditionally, SIN diminishes the unusually elevated expression of *α*7nAChR through the α7nAChR/ERK/Egr-1 feedback process, thus hindering M1 polarization in macrophages and reducing inflammation (94).

In the context of cerebral ischemia, SIN mitigates the inflammatory cascade triggered by ischemia, which is closely linked to inflammatory cytokines, inflammasomes, and inflammation-inducing enzymes. SIN protects neuronal cells by reducing the activation of microglia brought on by ischemia conditions and lowering inflammatory chemicals like IL-1β and TNF-α. This action is likely related to SIN’s ability to enhance IκBα protein expression, effectively inhibiting NF-κB signaling and regulating micro-glial inflammation (4, 95, 96). Moreover, SIN inhibits inflammasome activation during the initiation of CNS inflammation, targeting the NLRP3 inflammasome via an AMPK-dependent pathway and modulating astrocyte dopamine D2 receptors and the Alpha B-crystallin (CRYAB)/STAT3 axis to exert neuroprotective effects (97). SIN has also been shown to dose-dependently downregulate the expression of COX-2, a key inflammatory enzyme that facilitates prostaglandin synthesis and amplifies the inflammatory response (98). Notably, SIN inhibits the Src/FAK/P130Cas axis, reducing macrophage migration to inflamed regions and limiting the infiltration of peripheral macrophages into the brain, thus diminishing the overall inflammatory response (99). In a rat model of reversible arterial occlusion simulating cerebral ischemia, pre-treatment with SIN (90 mg/kg) via tail vein injection provided neuroprotection through an-ti-inflammatory effects, amelioration of acidosis, improvement of energy metabolism, and suppression of ASIC-1a levels (100). Likewise, in lab-based mouse models for brain ischemia and lack of oxygen–glucose, SIN mitigated conditions like cerebral infarction, edema, neuron death, and neurological impairments, simultaneously suppressing NLRP3 inflammasome activation and regulating neuroinflammation through the AMPK route (83, 97). These results collectively highlight SIN’s potent anti-inflammatory and neuroprotective properties.

### 5.2 Immunoregulation

Lymphocytes hold a pivotal position in the body’s immune reaction and are crucial in the adaptive immune system. Studies indicate that SIN has the ability to control proteins associated with the cell cycle and apoptosis, thereby simultaneously influencing the cell cycle and apoptosis in T cells and B cells. SIN also reduces inflammatory cell infiltration and mesangial cell proliferation, thus improving and preventing IgA nephropathy (101). Additionally, SIN has been reported to prevent the cell cycle from progressing from the G0/G1 phase to the S phase, as well as from G2/M phase, with its immunosuppressive activity on CD4^+^ primary lymphocytes mainly mediated by cystein-aspartate protease (Caspase-3)-mediated cell apoptosis. The expression levels of B-cell lymphoma-2 (Bcl-2) in activated T cells remain largely unchanged, indicating a potential non-involvement of Bcl-2 in SIN-triggered T cell apoptosis (102). SIN successfully reduced the levels of T-box transcription factor (T-bet), interferon-*γ* (IFN-γ), and IFN-γ/IL-4, T-bet/GATA-binding protein-3 (GATA3) in the decidua and serum of mice with recurrent spontaneous abortions, according to research using a mouse model of recurrent spontaneous abortion. However, it had no effect on the expression of GATA-3 and IL-4. Given that GATA-3 is a Th2 transcription factor and T-bet is a Th1 transcription factor, it follows that SIN significantly inhibits Th1 production. Alterations in T-bet/GATA-3 ratios indicate SIN’s function in maintaining the equilibrium between Th1 and Th2 (103).

Through various pathways, SIN suppresses the M1 polarization in macrophages, leading to a decrease in the production and secretion of diverse chemokines and pro-inflammatory cytokines, including TNF-*α* and IL-8 (89, 104). This action helps alleviate the damage of TNF-α, IL-8, and others to the immune system and improving macrophage phagocytosis and immune response. Further research indicates that the effects of SIN on macrophages are context-dependent. Specifically, SIN may inhibit α7nAChR expression through the regulation of the ERK1/2/Egr-1 pathway (92), thereby suppressing M1 polarization and promoting a shift toward the M2 phenotype (105, 106). This results in reduced secretion of M1-type cytokines (TNF-α, IL-1β, IL-6) and enhanced expression of M2-type cytokines such as Arginase-1 (Arg-1) and IL-10 via Nrf2 signaling (106), suggesting an immunoregulatory role in the ischemic brain. Conversely, in the immortalized murine macrophage-like cell line RAW264.7, SIN has been shown to induce apoptosis by activating ERK and altering the balance of Bcl-2 family proteins (107). These findings are not contradictory to those in T cells (102) but rather reflect cell type-specific responses: SIN-induced T-cell apoptosis proceeds via caspase-3–dependent mechanisms without Bcl-2 involvement, whereas in macrophages, apoptosis is mediated through ERK activation and modulation of Bcl-2 family proteins. Together, these studies highlight that SIN may exert dual roles in immune regulation depending on the cellular context and microenvironment. In RAW264.7 cells stimulated with LPS, Zhu et al. (91) demonstrated that SIN significantly inhibited the production of TNF-*α*, MCP-1, MIF, and MMP-9, downregulated CD14 and TLR4 expression, and reduced intracellular free calcium release. Mechanistically, SIN enhanced STAT3 phosphorylation, and this effect was attenuated by *α*7nAChR antagonists. Moreover, the JAK2 inhibitor AG490 diminished SIN’s inhibitory effect on TNF-α, indicating that SIN exerts anti-inflammatory actions by activating the α7nAChR/JAK2/STAT3 pathway. Consistent results have been reported in other studies, where SIN suppressed NF-κB activation and reduced the release of pro-inflammatory cytokines such as TNF-α, IL-1β, and IL-6 (108). In addition, Qin et al. (106) confirmed that SIN activates Nrf2 signaling, thereby downregulating M1-type cytokines while upregulating Arg-1, IL-10, and HO-1. Collectively, these findings indicate that SIN regulates multiple signaling pathways, including α7nAChR/JAK2/STAT3, NF-κB, and Nrf2, to inhibit macrophage pro-inflammatory activity. Through these mechanisms, SIN helps to maintain the dynamic balance between M1 and M2 macrophages, reduces macrophage chemotaxis and secretion, and inhibits macrophage apoptosis, thereby exerting potent immunosuppressive effects (Figure 4).

### 5.3 Antioxidant stress

ROS, emerging naturally from standard oxygen metabolism, are crucial in the process of cell signal transmission. Yet, when faced with environmental stress, ROS levels may swiftly rise, and oxidative stress denotes the harm resulting from the over-production of ROS in tissues and cells (8). Research indicates that SIN increases Bcl-2 levels and reduces the expression of bcl-2-associated X protein (Bax) and Caspase-3. SIN, through diminishing oxidative stress and obstructing the NF-κB signaling route, boosts anti-inflammatory abilities and adjusts associated cytokines, thus lessening liver dam-age caused by apoptosis and easing persistent lead poisoning (109). Within a mouse model of kidney fibrosis, SIN stimulated the Nrf2 signaling route, enhancing both the expression and function of enzymes involved in antioxidant and detoxification. This also disrupted the pro-fibrotic pathways of TGFβ/Smad and Wnt/β-catenin, altering pro-fibrotic protein levels in kidney cells treated with TGFβ and lessening renal fibrosis caused by one-sided ureteral blockage, suggesting SIN’s ability to suppress oxidative stress and safeguard the kidneys (110). Ramazi et al. (111) reported that a dose of 50 mg/kg SIN significantly restored levels of ROS, MDA, HO-1, and SOD in a temporal lobe epilepsy rat model, partially inhibiting the increase in NF-κB, TLR4, TNF-*α*, GFAP, and Caspase-1, although the effect on glutathione levels was not significant.

SIN mainly controls ROS generation via the Nrf2-related signaling route and is crucial in the development of chronic inflammatory conditions like rheumatoid arthritis. Under basal conditions, Nrf2 is bound to its inhibitor, Keap1. When activated by ROS or electrophilic substances, Nrf2 exits Keap1 and moves to the nucleus, functioning as a transcription factor to enhance the production of subsequent antioxidant and detoxi-fying enzymes like HO-1, NAD (P)H, NQO-1, SOD, and GSH-PX (112, 113). Examining SIN’s effects on a mouse model of *E. coli*-induced acute lung injury (ALI), Liu et al. (114) discovered that SIN greatly raised the protein expression of HO-1, Nrf2, and NQO-1, hence supporting Nrf2 nuclear translocation. By means of the Nrf2 signaling pathway, SIN reduces oxidative damage brought on by *E. coli*, as shown by these findings. Qin et al. (110) further showed that SIN alleviates oxidative stress induced by unilateral ureteral obstruction through the regulation of the Nrf2 signaling pathway, inhibit TGF-β-associated pro-fibrotic activity, and alleviate renal interstitial fibrosis. This suggests that SIN can suppress oxidative agents induced by TGF-β or H2O2 in renal cells and enhance detoxifying enzyme activity, such as SOD and GSH-PX, in both renal cells and fibrotic kidneys. It has also been confirmed that SIN phosphorylates p62 at the Ser351 site (corresponding to Ser349 in humans), leading to the degradation of Keap1 and upregulation of Nrf2 expression. SIN also promotes phosphorylation at the Thr269/Ser272 sites of p62, thereby activating the p62-Keap1-Nrf2 signaling pathway, exerting anti-arthritic effects (115).

In neuronal cells, SIN markedly enhances the production of antioxidant enzymes like HO-1 and SOD, affecting both protein and mRNA levels, thereby triggering the Nrf2 pathway (116, 117). This, in turn, reduces MDA levels in brain tissue, thereby enhancing neuronal resistance to oxidative stress. Furthermore, SIN decreases Caspase-9 expression, inhibiting cell apoptosis and protecting neural cells from oxidative stress-induced damage (118).

### 5.4 Pharmacological effects of SIN in other diseases

SIN has exhibited therapeutic efficacy in various diseases, including colitis (119), atherosclerosis (120), myocardial ischemia–reperfusion injury (100), chronic sciatic nerve injury (CCI) (121), renal injury (122), colorectal cancer (123), lung cancer, liver cancer, cervical cancer, bladder cancer, gastric cancer, and renal cancer (93, 124, 125). By downregulating pro-inflammatory factors including TNF-*α* and IL-6 and upregulating anti-inflammatory factors, SIN has anti-inflammatory actions that greatly improve experimental colitis (119). The anti-atherosclerotic effects of SIN (SIN) are attributed to its ability to inhibit inflammation progression, modulate immune cell functions, and suppress smooth muscle cell proliferation. Mechanistically, SIN reduces the expression of pro-inflammatory factors such as vascular cell adhesion molecule-1 (VCAM-1) and platelet-derived growth factor receptor-β (PDGFR-β) via multiple signaling pathways (120, 121, 126). B By blocking the TLR4/NF-κB signaling pathway, SIN successfully reduces oxidative stress and inflammation, reducing the atherosclerosis that rats get from a high-fat diet and vitamin D3 (127). SIN mitigates myocardial ischemia reperfusion injury by inhibiting myocardial cell apoptosis, oxidative stress, and reducing calcium ion levels, further alleviating arrhythmias caused by ischemia–reperfusion. SIN protects myocardial cells by increasing SOD system activity and clearing abnormal accumulation of ROS, and inhibition of NF-κB signaling pathway activation (128).

In a CCI rat model, SIN exerts dose-dependent analgesic effects, significantly alleviating heat hyperalgesia and mechanical allodynia by increasing withdrawal reflex latency and elevating pain thresholds (121). These effects are accompanied by reduced expression of proinflammatory cytokines (TNF-*α*, IL-1β, IL-6) and downregulation of RIP3, phosphorylated JNK, and c-Fos in the spinal cord, as well as an increased number of surviving neurons in the dorsal horn, indicating attenuation of CCI-induced neuronal death (121). The anticancer mechanisms of SIN primarily involve the regulation of NF-κB, tyrosine kinase, and STAT pathways, which inhibit the proliferation, invasion, and migration of tumor cells (93, 124, 125). Thioredoxin reductase (TrxR), an important component of the antioxidant system, is highly expressed in various malignant tumors and negatively correlates with patient prognosis, making it a potential novel target for cancer therapy (129). SIN is a novel TrxR inhibitor that effectively sup-presses TrxR activity, inducing excessive ROS production in tumor cells, mediating oxidative stress responses, and promoting tumor cell apoptosis (125). Additionally, SIN exhibits synergistic anticancer effects when combined with chemotherapeutic agents, such as 5-fluorouracil and cisplatin, potentiating their efficacy against multiple tumor types (120).

Beyond its role in inflammation and cancer, SIN also shows potential therapeutic efficacy in diseases such as diabetes, endotoxemia, asthma, cough, and depression. SIN has therapeutic effects in streptozotocin-induced gestational diabetes mellitus (GDM) rats with high safety, which is associated with the inhibition of inflammation and oxidative stress via the TLR4/MyD88/NF-κB signaling pathway (130). Moreover, SIN enhances pancreatic β-cell function and enhances insulin secretion, thereby preventing the occurrence of GDM and its related complications during pregnancy, offering a novel approach for the prevention and treatment of early diabetes. SIN inhibits dose-dependently the temperature elevation, cell adhesion, and systemic inflammatory responses induced by LPS in piglets, suggesting an anti-endotoxemia effect, though its main pathway requires further investigation (131). By inhibiting the activation of the TGF-β1/Smad3 signaling pathway and suppressing epithelial-mesenchymal transition, SIN significantly improves airway remodeling in an asthma mouse model, demonstrating bronchodilatory properties (132). On the other hand, SIN also exhibits therapeutic effects on chronic cough. It inhibits the expression of transient receptor potential vanilloid 1 (TRPV1) and decreases the expression of sex-determining region Y-box protein 5 (SOX5), thereby lowering intracellular Ca2 + concentration, reducing cough sensitivity, and alleviating chronic cough symptoms (133). Additionally, SIN exhibits antidepressant effects, significantly reversing the decrease of NE and serotonin levels in the hippocampus of mice subjected to chronic unpredictable mild stress, with improvements in depression-related symptoms (134). Further pharmacological effects of SIN are still to be explored.

## 6 Potential mechanisms of SIN in treating cerebral ischemia through the bone-brain Axis

Recent studies have indicated that there is bidirectional regulation between the bone and the brain, with communication occurring through inflammatory mediators, bone metabolic products, and the endocrine system. Research by Otto et al. (27) demonstrated that bioactive cytokines secreted by bones can enter the peripheral circulation and cross the BBB, thus modulating the function and metabolism of the CNS, playing a crucial central regulatory role. Ren et al. (135) also confirmed the existence of this communication pathway. Through this mechanism, bone-derived factors in circulation can regulate brain development and physiological processes. In particular, they support angiogenesis, improve synaptic plasticity, lower neuroinflammation, and pre-serve neuronal structure and function. Additionally, Herisson et al. (77) found vascular pathways connecting the brain and cranial bone marrow, which functioned as a route for bone marrow cell migration. According to this research, bone marrow cells and cytokines may have an impact on how brain disorders develop. In-depth studies have shown a close relationship between bone and cerebrovascular diseases. The occurrence of cerebrovascular diseases such as cerebral ischemia is often accompanied by BBB disruption, which facilitates the entry of bone marrow cells and bone-derived cytokines into the CNS, where they can have a variety of consequences. Moreover, bone marrow cavity injection can directly affect the bone-brain axis, thus providing a new approach and direction for drug delivery systems to act within the bone-brain axis, offering novel insights for the treatment of ischemic brain diseases. SIN itself has been reported to cross the blood–brain barrier and reach brain tissue after systemic administration. For example, Wu et al. (46) demonstrated that administration of SIN (10–30 mg/kg, i.p.) led to detectable levels in rat brain and significantly reduced infarct size in a middle cerebral artery occlusion (MCAO) model, via inhibition of acid-sensing ion channel 1a and L-type calcium channels. Moreover, SIN has ability to penetrate the CNS and exert neuroprotective outcomes *in vivo* (136). Cerebral ischemia triggers widespread activation of the peripheral immune system, releasing a large number of immune cells, inflammatory cytokines, and chemokines, which promote immune cell infiltration into the brain and further exacerbate brain injury (137). Therefore, inhibiting peripheral immune inflammatory responses is crucial in the treatment of ischemic brain diseases. Several studies have confirmed that intraarticular injection of SIN can effectively exert its pharmacological effects (138). SIN modulates M1 polarization of OCs through multiple mechanisms and promotes their polarization to the M2 type, thus maintaining the dynamic balance between the M1 and M2 subtypes of OCs. On one hand, SIN can lower the levels of pro-inflammatory M1 cytokines, while inhibiting the activity of NF-κB, NFATc1, MAPK, Ca2+, JAK/STAT, and other signaling pathways to suppress osteoclast differentiation, exerting immunosuppressive effects (139–141). Additionally, SIN can promote the secretion of anti-inflammatory M2 cytokines, which are bone-derived factors that can enter the peripheral circulation and cross the BBB, exerting anti-inflammatory effects at the site of brain injury (142). In addition to its immunomodulatory functions, accumulating evidence supports the direct neuroprotective role of SIN in the CNS. SIN attenuates amyloid β-induced astrocytic activation, reduce the release of ROS and inflammatory mediators, and protect both rodent and human neurons against indirect toxicity, suggesting its broader neuroprotective potential in neurodegenerative conditions (143). Beyond immunomodulation, experimental evidence has shown that SIN exerts direct neuroprotective effects in the CNS. In the same study by Wu et al. (144), sinomenine suppressed neuronal overactivation and reduced neuronal apoptosis in ischemic models via modulation of calcium channel activity. Also, recent comprehensive reviews summarizing multiple *in vivo* studies confirm SIN’s capability to inhibit microglial activation, reduce oxidative stress, and enhance neuronal survival after cerebral ischemia (136). Furthermore, Fu et al. demonstrated in a rat subarachnoid hemorrhage model that SIN administration improved neurological outcomes, attenuated brain edema and neuronal apoptosis, and suppressed microglial inflammatory responses via activation of the Nrf2/HO-1/NQO-1 signaling pathway (84).

**Figure 6:**
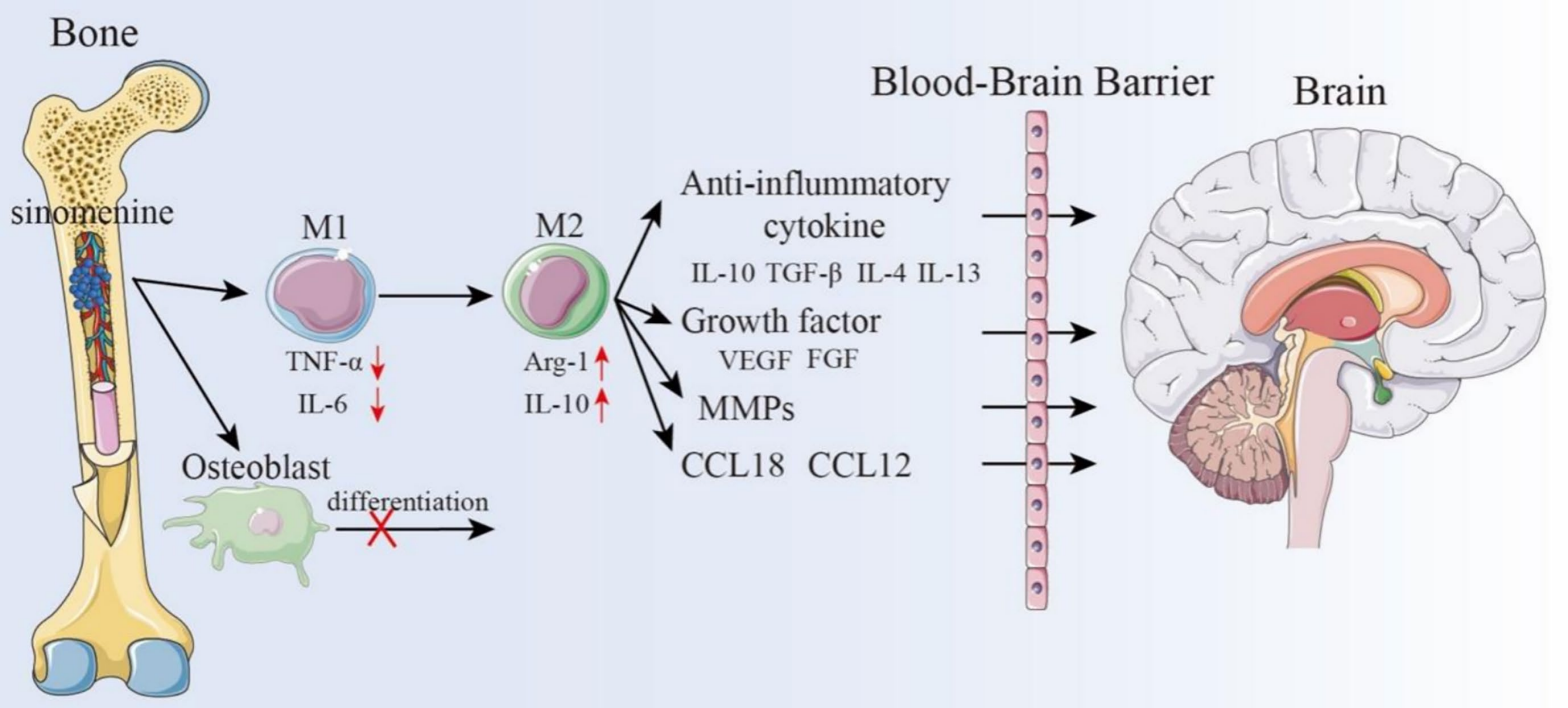
Potential mechanisms of SIN in treating cerebral ischemia through the bone-brain axis.

In addition, various cells in the bone marrow can migrate to the CNS and influence the progression of CNS disorders. With the aid of many receptors, integrins, selectins, and proteases, bone mesenchymal stem cells (BMSCs) can penetrate the BBB and go to the injured brain region (145). Nasal delivery of BMSCs can improve mental disorders in rats, significantly reduce infarct size, repair the BBB and neuro-vascular damage, and improve local cerebral blood flow in ischemic cortical areas. Furthermore, bone-derived cells can rescue social deficits in rats and promote the recovery of sensory-motor and olfactory functions (146). According to the emerging re-search, bone-derived macrophages may be crucial in the management of ischemic brain disorders. By utilizing the phagocytic, migratory, and targeting abilities of macrophages, they can serve as important drug delivery carriers (43), avoiding immune system phagocytosis, prolonging drug circulation time and half-life, and improving drug stability. Nanoparticle-based drug encapsulation is a promising strategy to enhance drug efficacy and minimize side effects. In this context, SIN has been shown to be capable of crossing the BBB (97, 144, 147), but its concentration in the brain is typically low. Liu et al. confirmed that 45 min after oral administration of SIN in rats, the drug was widely distributed across various organs, with the highest concentration observed in the kidneys, followed by the liver. In contrast, the drug concentrations in the brain and testes were relatively low (86). Huang et al. (85) also confirmed this finding. In addition, they observed a significant gender difference in the response of rats to sinomenine, with higher levels of SIN found in female rats compared to male rats. Currently, numerous studies have suggested that nanoparticle-mediated drug delivery may be a successful, non-invasive method of treating brain disorders (148). This strategy offers a novel avenue for SIN-targeted de-livery via the bone-brain axis, holding significant promise for ischemic brain therapy. Indeed, recent work in a chronic cerebral hypoperfusion (CCH) rat model showed that SIN promotes microglial polarization to the M2 phenotype and increases release of neuronal exosomes enriched in miRNA-223-3p, which when taken up by neurons inhibits NLRP3-mediated pyroptosis and improves cognitive function (149). In addition, dendrimer–SIN conjugates were demonstrated to selectively target activated microglia/macrophages in a rabbit model of pediatric traumatic brain injury, where they significantly attenuated acute neuroinflammation and oxidative stress compared with the free drug (150). These examples highlight that SIN can be delivered directly to the CNS via BBB-crossing carriers such as exosomes or dendrimer conjugates. Importantly, such direct CNS delivery does not negate the role of the bone–brain axis, which provides an additional, complementary pathway by which SIN can modulate systemic immunity and bone-derived signaling, ultimately influencing cerebral ischemia outcomes. Therefore, direct BBB-crossing delivery and bone–brain axis–mediated regulation should be considered parallel and complementary mechanisms, rather than mutually exclusive (Figure 6).

## 7 Advancements in novel dosage forms of SIN

The conventional preparation of SIN hydrochloride is unstable under light, heat and alkaline conditions, prone to decomposition, with a short-term biological half-life and low bioavailability. It also tends to promote the release of histamine, leading to adverse reactions such as rashes and gastrointestinal distress, which limits its wide-spread use in the management of the disease (4). To address these issues, scholars both domestically and internationally have combined pharmaceutical formulation theories with new technologies to improve the dosage form of SIN hydrochloride. Recent advancements have focused on the on new formulations such as SIN nanoparticles, exosomes, liposomes, microneedles, solute liquid crystal gels, and delivery systems, with the aim of exploring their application in the treatment of cerebral ischemia. Recent advancements in novel dosage forms of SIN are summarized in Table 2.

**Table 2:** Application of sinomenine nano-delivery system in the treatment of cerebral ischemia.

Nano-delivery system	Targeting strategy	Model (*In Vitro*/*In Vivo*)	Key parameters	Therapeutic outcomes	Proposed mechanism	Advantages	Year	Ref.
Nanoparticle	DSPE-PEG_2000_-HA	AIA rats	Size: 110.2 ± 12.35 nm, zeta potential: −12.65 mV	↓: Hind paw volume, arthritic score, rough bone surfaces, severe bone destruction of inflamed joints, synovial hyperplasia, inflammatory cell infiltration, joint space narrowing, cartilage destruction and bone erosion	↓: Activated macrophage, TNF-α, G-CSF, ROS, viability of HFLS-RA, inflammation	Controlled release, prolonged circulation half-life, specific arthritis-targeting abilit, escape from immune surveillance	2022	(151)
	Alendronate sodium	Collagen-induced arthritis rat model, RAW264.7 cells	Size: 396 ± 9.07 nm, drug loading content: 11.6 ± 1.3, drug loading efficiency: 60%	↓: Thickness of CIA rat paws, arthritis scores, degree of redness and swelling in rats	↓: ROS, MDA, IL-1β, TNF-α, p-p65, inflammation ↑: IL-10, Arg-1, M2 macrophages	Prolong the drug retention time inside the joint cavity, easy to synthesize, modify, and scale	2023	(152)
	HA-DSPE-PEG_2000_	AIA rats	Size: 115.00 ± 3.86 nm, load capacity: 103.10%, encapsulation capacity: 28.96%, zeta potential: −9.23 ± 0.23 mV	↓: Hind paw volume, arthritic score, synovial inflammation, cartilage loss, chondrocyte count, loss of the cartilage matrix, rough joint surfaces, prominent marginal osteophytes, narrowed joint cavities, impaired interphalangeal and metatarsal joint correspondence	↓: TNF-α, IL-6, the viability of RAFLS, proliferating cells, cyclin B1, IL-6, TNF-α, PI3K/Akt, SGK, cell cycle pathway ↑: M2 macrophages, IL-10, FoxO, cell apoptosis	Precise targeting of the synovial lesion site in arthritis	2024	(153)
	D-Sino conjugates	Traumatic brain injury rabbits model	Size: 4.87 ± 0.43 nm, zeta potential: −0.16 ± 0.17 mV		↓: TNF-α, NO, IL-1β, CCL-3, IL-1β, IL-6, iNOS, NF-κB activation and its nuclear translocation	Cross BBB, specifically targeted activated microglia/macrophages at the site of injury in the brain, increasing the therapeutic window	2020	(150)
Exosomes		HepG2 and L02 cells	Average diameter: 84 ± 3 nm, loading efficiency: 17–19%		↑: Cell cycle arrest, cellular apoptosis ↓: migration of HepG2 cells, CD44	Improve the bioavailability of SIN and enhance the anti-tumor efficacy of SIN	2021	(155)
Hybrid exosomes		Collagen-induced arthritis rats	Size: 132.70 ± 4.07 nm, Zeta potential: −17.90 ± 2.13 mV, encapsulation rate: 48.21% ± 3.12%, drug loading: 3.17% ± 0.36%	↑: Microvascular comprehensive score, vascular resistance ↑: general condition, swelling degree of foot, arthritis index and immune organ index	↓: TNF-α, IL-6	More stable and durable drug effect, prolonged the half-life of drugs	2017	(156)
Liposomes	Passive targeting, microwave hyperthermia	RA model rats	Size: 100 nm EE%: 90% or more	↓: Thickness of the paw, arthritic scores, destruction, bone loss, synovial inflammation, pannus formation, bone erosion	↓: TNF-α, IL-6	Effectively taken up by lipopolysaccharide-activated HUVECs	2020	(159)
Ethosomes	Topical application	Xylene-induced mouse ear edema model	Size: 157.08 ± 11.72 nm cumulative amount for 24 h: 663.8 ± 27.4 μg/cm^2^	↓: Xylene-induced ear edema		Enhance transdermal permeation of SH, strong anti-inflammatory activity on the xylene-induced ear edema	2016	(161)
Transfersomes		New Zealand rabbits, Male sprague dawley rats	Size: 109.10 ± 1.80 nm, PDI: 0.156 ± 0.007, Zeta potential: −18.90 ± 2.21, Elasticity: 24.50 ± 0.50, EE: 22.38 ± 0.79			Better in vitro skin permeation property than conventional liposomes, high trandermal drug delivery rate, excellent transdermal drug-delivery carriers, good percutaneous permeation property	2017	(166)
	Topical application	New Zealand rabbits, Male sprague dawley rats	Size: 102.00 ± 1.05 nm, PDI: 0.090 ± 0.029, Zeta potential: −8.37 ± 0.40, Elasticity: 40.84 ± 1.01, EE: 20.12 ± 1.63			Penetrate deeply into the skin layers, deliver more drug to joint cavities and less to blood	2020	(167)
	Transdermal administration	New Zealand rabbits, Male sprague dawley rats	Size: 83.31 ± 0.08 nm, PDI: 0.062 ± 0.017, Zeta potential: −32.57 ± 3.27, EE: 39.82 ± 0.97			Penetrate through stratum corneum into deeper epidermis, broke down rapidly and released SH after entering the deep parts of the skin	2022	(168)
Microneedles	Transdermal drug delivery	Male sprague dawley rats	Length: 0.5 mm, center-to-center spacing: 1.0 mm, in skin: *T*_1/2*α*_: 188.54 ± 5.16, *T*_1/2β_: 99.48 ± 3.65, Tmax: 240, *C_max_*: 10.80 ± 0.43, AUC_0–t_: 2378.97 ± 58.68, MRT_0–*t*_: 272.84 ± 1.15, in blood: *T*_1/2*α*_: 108.49 ± 8.91, *T*_1/2β_: 383.98 ± 6.51, *T_max_*: 300, *C_max_*: 6.80 ± 0.26, AUC_0–*t*_: 1327.53 ± 11.73, MRT_0–*t*_: 306.68 ± 1.37			Increased the cumulative permeability and permeability rates, enhanced the percutaneous penetration effect of SH	2020	(174)
	Transdermal administration	Wistar rats	Length: 500 μm, distance: 1 mm, permeation rate: 129.29 ± 7.52 μg cm^−2^ h^−1^, accumulation osmolality: 5567.48 ± 206.57 μg cm^−2^,			Provide a longer retention time in blood for SH, lower clearance, longer retention time, higher bioavailability and stability	2016	(175)
	Transdermal administration	Wistar rats	Interspacing of needles: l.0 mm, height: 600 μm, diameter at the base: 300 μm, weighted: 1.20 ± 0.01 g			Transdermal absorption of SH was enhanced and sustained	2017	(176)
	Transdermal administration	New Zealand rabbits	Length: 0.8 mm, base diameter: 0.3 mm			Enhanced bioavailability and permeability of SH	2015	(177)
Hexagonal liquid crystalline system	Transdermal applications	Sprague–Dawley rats	Hexagonal phases			Penetration-promoting effect for transdermal applications in SH	2022	(179)
Hydrogel	Topical application	Atopic dermatitis model	Size: 133.8 ± 14.5, viscosity: 9.83 ± 0.11 mPa·s, pH: 7.27 ± 0.01, encapsulation rate: 72.27%	↓: Ear Swelling, Epithelial keratinization, hyperplasia of the spiny layer, inflammatory infiltrate around the blood vessels in the dermis ↑: structure of each layer was more complete with clear borders	↓: Hydroxyl radicals	Slow and controlled release, stable properties, good dispersion, an excellent dermal penetration effect	2024	(180)
		Kunming mice	Viscosities: 12.6679 mPa, drug loading: 1.98 ± 0.01 mg/mL	↓: DPPH radicals, H_2_O_2_, MDA		Good sustained-release and antioxidant effects	2022	(181)
	LPS-induced RAW264 cells	OA model	Sol–gel transition temperature: 21.8 °c, SMN release after 48 h: 44.72 ± 7.83%	↑: Trabecular thickness, bone volume-to-tissue volume ratio ↓: osteophyte formation, knee joint pain, trabecular separation, IL-1β	↑: Chondrocyte proliferation, M2 macrophage polarization, ↓: apoptosis, MMP3, ROS, MMP-13,	Controlled release capabilities, prolonged retention in the synovial fluid	2025	(182)
In situ gel	Topical administration	Experimental autoimmune uveitis animal models	Drug release: 96.3%, over a period of 480 min	↓: Inflammatory response	↓: Inflammatory cells	The percorneal retention was improved from 10 min to 25 min, enhance bioavailability through its longer elimination time and the ability to sustain drug release	2013	(183)
In situ hexagonal liquid crystal	Injected into the left knee joint of each rat	AA rats	Cumulative SMH release of 99.9% within 240 h,	↓: Synovial hyperplasia, inflammatory cell infiltration	↓: IL-1β	Significantly reduced the leakage of SMH into systemic circulation,	2019	(186)

### 7.1 Nanoparticles

Nanoparticles, materials with at least one dimension on the nanoscale, with properties such as high surface area, easy surface modification, strong stability, high encapsulation efficiency, targeted delivery, and the ability to simultaneously deliver therapeutic agents, have attracted great attention in the field of biomedical applications. Prussian blue nanoparticles (PBNPs) have emerged as promising drug carriers because of high permeability and strong biocompatibility, and can serve as drug carriers for targeted drug delivery, release control, bioavailability, and dosage reduction. The cell membrane after binding with nanoparticles can escape immune phagocytosis, further enhancing the biological functions of the drug delivery system, such as prolonged circulation and active targeting. To treat rheumatoid arthritis, multifunctional nanoparticles of hydrochloride SIN (HA@M@PB@SINNPs) was designed, using hyalu-ronic acid (HA) as a targeting molecule, with the drug loaded onto PBNPs to form PB@SINNPs The red blood cell membrane and macrophage membrane were fused by sonication and subsequent magnetic stirring at 37 °C to form hybrid vesicles, which were then used to disguise PB@SINNPs (151). *In vitro*, HA@M@PB@SINNPs inhibited fibroblast-like synoviocyte proliferation by scavenging ROS and suppressing the secretion of pro-inflammatory cytokines. *In vivo*, in an AIA rat model, the circulation half-life of HA@M@PB@SINNPs at the site of arthritis reached 6.51 h, which is eight times that of free sinomenine hydrochloride (SH), significantly enhancing drug accumulation. This study demonstrates that HA@M@PB@SINNPs are excellent carriers for controlling the liberation and targeted accumulation of SIN in rheumatoid synovial joints. Shang et al. constructed a SIN-loaded nanomedicine with HA as the backbone, which had subchondral bone ad-sorption ability, prolonged residence time in inflamed joints and responsiveness to ROS (152). In brief, the amine-functionalized polyethylene glycol (PEG), alendronate (Ald), and ethyl L-methionine (Met) were sequentially coupled with the carboxyl group of HA through amination, followed by electrostatic and hydrophobic interactions to self-assemble with Sin, forming the PAM-HA@Sin NPs. PAM-HA@Sin NPs effectively prolonged the retention time of the drug in the joint cavity. The therapeutic PAM-HA polymer carrier could increase joint lubrication and reduce oxidative stress. At the same time, pro-inflammatory factors (TNF-*α* and IL-1β) were downregulated and anti-inflammatory factors (Arg-1 and IL-10) were upregulated due to SIN produced through the NF-κB pathway, which decreased M1 macrophage levels and increased M2 macrophage levels. Lin et al. (153) developed a biomimetic nanomedicine system targeting synovial macrophages and FLS. This system loaded SIN onto graphene oxide quantum dots (GOQDs) and combined them with hybrid membranes incorporating HA to construct a novel nanomedicine system named HA@RFM@GP@SIN NPs. HA@RFM@GP@SIN NPs promoted the transition from M1 to M2 macrophages and inhibited the abnormal proliferation of FLS *in vitro*. Notably, it was demonstrated that HA@RFM@GP@SIN NPs had anti-arthritic effects via interfering with the PI3K/Akt/SGK/FoxO pathway, ovarian steroidogenesis, steroid biosynthesis, and the metabolism of tryptophan and tyrosine. In another study, SIN was combined with hy-droxyl-terminated fourth-generation PAMAM dendrimers to prepare nanoparticles (D-Sino) to alleviate early inflammation in traumatic brain injury (TBI). D-Sino in-creased the uptake of dendrimers by 1.8 times, enhancing the intracellular bioavailability of Sino. D-Sino complexes alleviated early/acute inflammation in mouse macro-phages by inhibiting pro-inflammatory cytokines (TNF-*α*, IL-1β, CCL-3, and IL-6), effectively preventing LPS-induced NF-κB activation and nuclear translocation, and targeting activated microglia in the brain injury domain, significantly reducing pro-inflammatory microglial activation (150).

### 7.2 Exosomes

Exosomes are vesicles secreted by various cells that contain bioactive substances. They have a diameter of 30–100 nm and possess a negatively charged phospholipid bi-layer structure (154). Due to their excellent biocompatibility, targeting ability, and high stability, they can serve as a potentially effective drug delivery system. Using differential centrifugation, exosomes were isolated from the plasma of SD rats and loaded with SIN, resulting in Exo-SIN with a particle size of 90 ± 4 nm (155). Exo-SIN was shown to inhibit the migration of HepG2 liver cancer cells, induce cell cycle arrest, and promote apoptosis in a dose-dependent manner. Exo-SIN was able to continuously release over a period of 48 h at pH 7.4 and 37 °C, with a cumulative release amount reaching 86.15%, significantly improving the bioavailability of SIN and extending its duration of action. Additionally, milk exosomes were fused with liposomes to create a mixed exosome formulation loaded with SIN, with milk exosomes used as a control group (156). The mixed exosome formulation demonstrated improved performance, with higher encapsulation efficiency (48.21% ± 3.12%) and drug loading (3.17% ± 0.36%) compared to the milk exosomes alone (31.64% ± 2.48 and 2.35% ± 0.52%, respectively). Compared to the control group, the mixed exosomes demonstrated more stable and sustained drug effects while also successfully enhancing the drug loading and biocompatibility of the liposomes.

### 7.3 Liposomes

Liposomes exhibit excellent biocompatibility, which can reduce irritation and side effects of drugs, and are commonly used in transdermal drug delivery. SIN encapsulated in liposomes for local delivery can concentrate SIN at the target site, reducing systemic absorption-related side effects. Traditional liposomes, made from materials such as soybean phospholipids, cholesterol, and vitamin E, can be used to encapsulate SIN. In recent years, stimulus-responsive liposomes have attracted considerable attention, as modifying liposomes allows for more precise control over the timing and location of drug release, enhancing drug delivery and dosage control, thus improving therapeutic efficacy (157, 158). pH gradient methods were successfully used to prepare SIN hydrochloride thermosensitive liposomes (SIN-TSL). These liposomes were composed of dipalmitoyl phosphatidylcholine, hydrogenated soy lecithin, and cholesterol, with an encapsulation efficiency of up to 90% and a particle size of approximately 116.3 ± 5.03 nm, showing high drug loading. Thermosensitive liposomes exhibited stronger targeting capabilities than conventional liposomes. SIN-TSL could release the drug efficiently and controllably at the RA rat hind limb for 48 h, extending the drug’s half-life (159). A novel transdermal drug delivery system for SIN, based on fatty acid-arginine vesicles ([Arg][Dec]), was also developed to enhance SIN’s transdermal absorption (160). Importantly, the centrifugal stability (CS) improved to 90.4% and the encapsulation efficiency of SIN in [Arg][Dec] vesicles rose to 83.5%. In addition, *in vitro* release studies showed that SIN rapidly released into the water within the first 3 h, with the cumulative release rate exceeding 95% after 17 h. In transdermal experiments, the cumulative permeability of [Arg][Dec] vesicles was 1665.59 μg·cm^−2^, significantly higher than the 663.8 μg·cm^−2^ reported in the literature (161). Traditional liposomes, due to their larger particle size, high manufacturing costs, and low stability, are less efficient in penetrating the skin. Ethosomal liposomes, a novel type of liposome containing a high concentration of ethanol (162), can deliver SIN via the stratum corneum to reach deeper skin layers and even the bloodstream (161). Yan et al. (161) prepared a novel SIN-loaded ethosome (SE) by injection. Within 24 h, the cumulative transdermal flux of SIN in SE was 663.8 μg/cm^2^, with a deposition of 18.5 μg/cm^2^, whereas the cumulative transdermal flux of SIN in an ethanol-water solution was only 329.2 μg/cm^2^ with a deposition of 5.2 μg/cm^2^, indicating that SE significantly improved SIN’s transdermal performance. In a xylene-induced mouse ear edema model, SE showed significant inhibition of ear edema (30.01%), significantly higher than the inhibition rate of SIN-HCl ethanol-water solution (20.83%).

Transfersomes (TFs) are a novel class of lipid vesicles made from edge activators and phospholipids. Edge activators cause lipid vesicles’ bilayer membrane to become less stable, become more deformable, and facilitate vesicles’ passage through the skin’s micropores while avoiding the stratum corneum barrier, which is the primary barrier and rate-limiting step for drug diffusion through the skin (163, 164). Furthermore, TFS can increase drug deposition in the skin and extend the duration of effective drug concentrations, reducing the frequency of administration (165). Conventional liposomes and sodium deoxycholate edge-activated transfersomes (DTFS) served as controls whereas mixed monoterpenes were employed as edge activators to create mixed monoterpenes edge-activated PEGylated transfersomes (MMPTs) (166). In *in vitro* skin permeation studies, the cumulative skin permeation of SIN in the optimized TFSs3 formulation was 1.5 times higher than DTFS and 3 times higher than conventional liposomes. This indicates that MMPTs exhibited better skin permeation properties than traditional liposomes. In *in vivo* pharmacokinetic studies, the steady-state concentration (Css) and AUC0 → t increased, while the MRT0 → inf was shortened, suggesting that MMPTs had a higher transdermal drug delivery rate. The distribution of MMPTs in various skin layers and the pharmacokinetics of SIN in the blood and joint cavity were examined in another study utilizing MMPTs using confocal laser scanning microscopy (CLSM) and dual-site micro dialysis in conjunction with LCMS/MS (167). The results showed that, compared to other types of vesicles, a moderate number of mixed mono-terpenes significantly increased the elasticity of MMPTs. CLSM analysis indicated that LPS was confined to the stratum corneum, while MMPTs were mainly localized in the deeper skin layers, indicating their potential to facilitate transdermal delivery of SIN. Furthermore, LC–MS/MS analysis showed that, in the joint cavity, the steady-state concentration (Css) and the area under the concentration–time curve (AUC0 → t) of SIN delivered by MMPTs were 2.1- and 2.5-fold higher, respectively, than those achieved with LPS. In contrast, in the blood, the Css and AUC0 → t of SIN in MMPTs were ap-proximately one-third of those in LPS. Therefore, MMPTs also enhanced SIN delivery to the joint cavity. Sodium deoxycholate was used as an edge activator to prepare SIN hydrochloride transfersomes (SHTs), with SH liposomes (SHLs) prepared as a control formulation (168). In *in vitro* permeation tests, after 36 h of administration, the cumulative permeation and cumulative permeation rate of SH in SHTs were ap-proximately 1.7 times those in SHLs. Furthermore, compared to SHLs, SHTs exhibited a 62% higher deposition percentage (Qp) in the subcutaneous skin layer beneath the stratum corneum. The Css and AUC0 → t of SHTs were roughly 8.8 and 8.0 times those of SHLs, respectively, according to pharmacokinetic data. According to blood pharmaco-kinetic data, SHTs’ Css and AUC0 → t were roughly 3.7 and 2.9 times greater than SHLs’, respectively. These experiments confirmed that TFS demonstrated superior transdermal penetration compared to conventional liposomes.

### 7.4 Microneedles

Microneedles can overcome the skin barrier and deliver drugs trans dermally with minimal invasiveness (169–172). Microneedles consist of multiple micro-projections less than 2 mm in height and, due to their direct action on the lesion, can reduce the dosage and enhance safety, playing a vital role in transdermal drug delivery systems (173). A dissolving microneedle (SH-MN) loaded with SIN was fabricated using a casting method from polyethylene pyrrolidone and chondroitin sulfate (174). SH-MN showed higher cumulative permeation and permeation rates than SH-G, with cumulative permeation and permeation rates of 5.31 and 5.06 times, respectively. In the skin and blood, the area under the curve (AUC) for SH-MN was 1.43 and 1.63 times that of SH-G, respectively, indicating that microneedles enhanced SIN’s transdermal penetration and increased its bioavailability (175). Pharmacokinetic analysis further revealed that the maximum plasma concentration (Cmax) of SIN in the microneedle group (0.74 ± 0.13 mg/mL) was more than twice that of the hydrogel group (0.33 ± 0.029 mg/mL). The time to reach maximum concentration (Tmax) was significantly prolonged in the microneedle group (18.24 ± 5.26 h) compared with the hydrogel group (7.32 ± 0.21 h), suggesting a more sustained release profile. After 4 h, the microneedle tips had almost completely dissolved, and the drug concentration in the skin reached its peak, with a subcutaneous penetration depth of 200 μm. A microneedle array made of polyvinyl alcohol (PVA) and maltose (MT) showed better performance compared to SH-loaded hydrogel (175). The MT/PVA microneedle array loaded with SH demonstrated lower clearance, longer retention time, higher bioavailability, and better stability. Another study used liquid crystal (H2)-composite dissolving microneedles (DM) for transdermal delivery of SH (176). Compared to other control groups, the composite DM enhanced and maintained SH’s transdermal delivery, with a significantly increased cumulative permeation. Wu et al. prepared dissolving microneedles (SH-DM) using MT and poly (lactic-co-glycolic acid) (PLGA) copolymer as materials (177). During the *in vivo* transdermal study in rabbits, SH-DM was administered at a dose of 50 mg/kg by pressing the microneedle array (7 × 10) into the shaved dorsal skin for 20 s, while SH-G was applied topically at the same dose. The results showed that SH-DM exhibited a 1.99-fold higher AUC compared to SH-G, with a relative bioavailability of 199.21%, indicating that SH-DM significantly enhanced the bioavailability and permeability of SH (177).

### 7.5 Gels

Gels are semi-solid drug delivery carriers with a three-dimensional network structure. They exhibit high drug loading capacity and are suitable for lipophilic, hydrophilic, and amphiphilic drugs, making them a promising novel transdermal drug delivery system (178). A hexagonal phase liquid crystal gel was prepared to co-load the hydrophilic drug SIN hydrochloride and the lipophilic drug cinnamaldehyde. Small-angle X-ray scattering, rheological techniques, laser scanning confocal fluorescence microscopy, and *in vitro* release studies showed that the addition of an appropriate amount of lipophilic drug to the hexagonal phase liquid crystal gel enhanced the permeability of the hydrophilic drug while slowing down the release of both drugs, thereby prolonging the drug retention time and improving local bioavailability (179). SIN hydrochloride liposomes (SINH-L) were uniformly dispersed in a hyaluronic acid gel matrix to form SINH-LH, with an encapsulation efficiency of 72.27% (180). SINH-LH demonstrated good transdermal effects, enabling slow drug release while maintaining moisture at the application site, and exhibited strong antioxidant proper-ties with notable scavenging abilities against hydroxyl and ABT free radicals. Another study also confirmed the antioxidant effect of SIN-loaded liposomal hydrogel, which also showed some ability to clear MDA in ex vivo organ homogenates (181). Sheng et al. (182) developed a hydrogel system carrying SIN-loaded cerium dioxide nanoparticles (SMN-CeO₂@G). Within 48 h, the cumulative release of SIN from SMN-CeO₂@G was 44.72 ± 7.83%. At a concentration of 0.5 μg/mL, SMN-CeO₂@G promoted proliferation and reduced apoptosis of ATDC5 chondrocytes while decreasing IL-1β-induced MMP-13 secretion. It also reduced ROS levels in chondrocytes by promoting macrophage M2 polarization. A single intra-articular injection of SMN-CeO₂@G significantly reduced osteophyte formation, normalized subchondral bone, alleviated pain sensitivity, and lowered serum levels of IL-1β and MMP-13 in an osteoarthritis model. Song et al. (183) developed and optimized an *in situ* gel of SIN hydrochloride for the treatment of uveitis. The optimal formulation, F2-3, containing 0.5% SIN, released 96.3% of its drug content within 480 min. Pharmacokinetic studies showed that the AUC 0-t and C_max values for the aqueous humor in the gel group were 2.70 times and 1.79 times higher, respectively, than those in the control group. Lyotropic liquid crystals (LLC), formed by the self-assembly of amphiphilic molecules in water into highly ordered lamellar and non-lamellar phases, including hexagonal phase and cubic phase liquid crystals, can control drug release and enhance drug solubility, thereby maintaining a more stable drug concentration (184, 185). An in situ hexagonal liquid crystal filled with SIN hydrochloride for intraarticular injection was used to create a unique medication delivery method. In order to improve patient compliance, this system can stay in the joint cavity for more than seven days, minimizing systemic exposure and lowering the need for frequent injections (186).

## 8 Discussion and conclusion

Cerebral ischemia remains a major global health challenge due to its high rates of morbidity, mortality, and long-term neurological disability. The complex pathological cascade, including neuroinflammation, oxidative stress, blood–brain barrier disruption, and glial cell activation, critically contributes to irreversible brain damage. At present, therapeutic strategies for ischemic stroke primarily involve thrombolysis with tissue plasminogen activator and mechanical thrombectomy procedures. However, the narrow therapeutic time window greatly limits both the efficacy and safety of these interventions (187). In addition, a study has shown that certain surgical procedures fail to improve patient recovery and may even be linked to worse outcomes, including higher mortality and functional impairment (188). Consequently, there is an urgent need to explore alternative, more effective therapeutic approaches, making the development of novel pharmacological agents for stroke an imperative priority. Notably, some compound drugs have already shown encouraging results in this field; for instance, studies have indicated that DL-3-n-butylphthalide together with Edaravone dexborneol can enhance neurological performance and mitigate cognitive deficits in experimental models of ischemic stroke by jointly inhibiting inflammatory responses and oxidative stress (189, 190). This review highlights the multifaceted neuroprotective potential of SIN, a bioactive alkaloid with well-documented anti-inflammatory, antioxidant, and immunomodulatory properties.

However, despite promising preclinical results, the clinical translation of SIN is limited by its poor pharmacokinetic profile, including low oral bioavailability and rapid systemic clearance. Advances in nanotechnology-based drug delivery systems, such as microneedles, hydrogels, and liposomal formulations, have demonstrated significant improvements in SIN’s stability, brain-targeting capacity, and therapeutic efficacy. These novel delivery strategies not only enhance drug bioavailability but also offer sustained release and improved patient compliance, which are essential for effective treatment of cerebral ischemia.

An emerging focus of this review is the bone–brain axis, a bidirectional communication pathway that has recently garnered attention for its potential in modulating neurological diseases. Bone-derived cytokines, osteocalcin, and other mediators can cross the BBB and influence CNS functions, offering a unique route for targeted drug delivery. Leveraging this axis for SIN administration could represent a transformative approach, potentially enhancing therapeutic concentrations at the ischemic brain sites while minimizing systemic side effects.

Furthermore, the proposed bone marrow cavity injection-based delivery system may selectively modulate microglial polarization by suppressing the pro-inflammatory M1 phenotype and promoting the anti-inflammatory M2 phenotype, thereby facilitating neuroprotection. The interaction between microglia and astrocytes, particularly the activation of neuroprotective A2 astrocytes, could further contribute to mitigating neuroinflammation and restoring neural homeostasis.

A potential point of contention lies in the possibility that if drugs can cross the BBB and act directly within the CNS, the regulatory influence of the bone–brain axis may be relatively diminished. This issue can be approached by considering the distinct mechanisms of action of the two strategies. The bone–brain axis may play a more critical role in chronic or long-term regulation. On the one hand, it alleviates neuroinflammation by modulating immune responses; on the other hand, bone-derived cells and metabolites are capable of crossing the BBB, thereby providing sustained immune regulation and neuroprotection. In contrast, drugs that act directly on the CNS—for instance, via nanoparticle-based delivery systems capable of penetrating the BBB—can exert more rapid neuroprotective effects during the acute phase. Such approaches enable direct delivery of therapeutic agents to ischemic regions, alleviating brain tissue injury within a short time frame. Nevertheless, these two mechanisms are not mutually exclusive but rather complementary. The bone–brain axis provides continuous immune modulation and long-term neuroprotection, whereas direct CNS drug delivery offers immediate mitigation of acute brain injury. The integration of these strategies has the potential to markedly enhance therapeutic efficacy, particularly in ischemic brain injury, by improving outcomes across both the acute and chronic phases.

Nevertheless, several challenges remain before clinical application can be realized. The precise mechanisms governing the bone–brain axis-mediated delivery and SIN’s interaction with neural and immune cells require further elucidation. Additionally, comprehensive *in vivo* studies and well-designed clinical trials are needed to validate the safety, efficacy, and optimal dosing regimens of SIN nanoformulations targeting the bone–brain axis.

In summary, SIN can improve the inflammatory microenvironment through various mechanisms, inhibit neuronal damage, alleviate symptoms of cerebral ischaemia and improve patients’ quality of life. However, due to the inherent limitations of traditional Chinese medicine, we believe that the combination of novel dosage forms with traditional drug delivery systems can reduce the required dosage of SIN, improve its targeting ability and provide new approaches for the treatment of cerebral ischemia. We propose a bone marrow cavity injection-based drug delivery system designed to suppress M1 microglial polarization while promoting M2 polarization, thereby facilitating the release of anti-inflammatory factors and reducing neuronal damage. In addition, given the ‘cross-talk’ between microglia and astrocytes, this system could potentially activate A2 reactive astrocytes, which may help limit the excessive production of pro-inflammatory factors and oxidative stress molecules, ultimately restoring neural homeostasis. In conclusion, as a widely used traditional medicine, further research on SIN will be of significant clinical value and may contribute to the development of advanced therapeutic approaches.

## Glossary

SIN: Sinomenine
CNS: Central nervous system
ATP: Adenosine triphosphate
DAMPs: Damage associated molecular patterns
HMGB1: High-mobility group box 1
HSPs: Heat shock proteins
BBB: Blood–brain barrier
NF-κB: Nuclear Factor kappa B
AP-1: Activator Protein 1
TNF-α: Tumor necrosis factor-α
IL-1β: Interleukin-1β
COX2: Cyclooxygenase-2
MMP: Matrix Metalloproteinases
ZO-1: Zonula Occludens-1
MAPK: Mitogen-activated protein kinase
GFAP: Glial fibrillary acidic protein
NLRP 3: NOD-like receptor thermal protein domain associated protein 3
PAMP: Pathogen-associated molecular pattern
ROS: Reactive oxygen species
Aβ: Amyloid-beta
RANKL: Receptor activator of nuclear factor-κB ligand
NFATc1: Nuclear factor of activated T-cells cytoplasmic 1
BMPs: Bone morphogenetic proteins
TGF-β: Transforming growth factor-β
OBs: Osteoblasts
OCs: Osteoclasts
MDA: Malondialdehyde
SOD: Superoxide dismutase
GSH-PX: Glutathione peroxidase
PHD1: Prolyl hydroxylase domain-containing protein 1
NE: Norepinephrine
SNS: Sympathetic nervous system
NPY: Neuropeptide Y
CART: Cocaine-amphetamine regulated transcript
5-HT: Serotonin
NPU: Neuropeptide U
PNS: Parasympathetic nervous system
Ach: Acetylcholine
nAChRs: Nicotinic acetylcholine receptors
VIP: Vasoactive intestinal peptide
CGRP: Calcitonin gene-related peptide
GABA: *γ*-aminobutyric acid
GHRP: Growth hormone releasing peptide
CRF: Corticotropin-releasing factor
AD: Alzheimer’s disease
GPCR: G protein-coupled receptor
ASR: Acute stress response
FGF23: Fibroblast growth factor 23
BDNF: Brain-derived neurotrophic factor
IGF-1: Insulin-like growth factor 1
GDF-15: Growth differentiation factor 15
Treg: Regulatory T cell
H₂O₂: Hydrogen peroxide
LPS: Lipopolysaccharide
FLS: Fibroblast-like synoviocytes
AIA: Adjuvant-induced arthritis
α7nAChR: α7 nicotinic acetylcholine receptor
CAP: Cholinergic anti-inflammatory pathway
Nrf2: Nuclear factor erythroid 2-related factor 2
HO-1: Heme oxygenase-1
TLR4: Toll-like receptor 4
JAK2: Janus kinase 2
STAT3: Signal transducer and activator of transcription 3
ERK: Extracellular signal-regulated kinase
Egr-1: Early growth response 1
CRYAB: Alpha B-crystallin
Caspase-3: Cystein-aspartate protease
Bcl-2: B-cell lymphoma-2
T-bet: T-box transcription factor
IFN-γ: Interferon-γ
GATA3: GATA-binding protein-3
Arg-1: Arginase-1
MyD88: Myeloid differentiation factor 88
Bax: Bcl-2-associated X protein
NAD(P)H: Nicotinamide adenine dinucleotide
NQO-1: Quinone dehydrogenase-1
ALI: Acute lung injury
VCAM-1: Vascular cell adhesion molecule-1
PDGFR-β: Platelet-derived growth factor receptor-β
TrxR: Thioredoxin reductase
GDM: Gestational diabetes mellitus
SOX5: Sex-determining region Y-box protein 5
TRPV1: Transient receptor potential vanilloid 1
MCAO: Middle cerebral artery occlusion
BMSCs: Bone mesenchymal stem cells
CCH: Chronic cerebral hypoperfusion
PBNPs: Prussian blue nanoparticles
HA: Hyaluronic acid
SH: Sinomenine hydrochloride
PEG: Polyethylene glycol
Ald: Alendronate
Met: Ethyl L-methionine
GOQDs: Graphene oxide quantum dots
TBI: Traumatic brain injury
SIN-TSL: Sinomenine hydrochloride thermosensitive liposomes
CS: Centrifugal stability
SE: SIN-loaded ethosome
TFs: Transfersomes
MMPTs: Mixed monoterpenes edge-activated PEGylated transfersomes
DTFs: Deoxycholate edge-activated transfersomes
C_ss_: Steady-state concentration
CLSM: Confocal laser scanning microscopy
SHTs: Sinomenine hydrochloride transfersomes
SHLs: SH liposomes
AUC: Area under the curve
PVA: Polyvinyl alcohol
MT: Maltose
DM: Dissolving microneedles
PLGA: Poly(lactic-co-glycolic acid)
SINH-L: Sinomenine hydrochloride liposomes
